# Metabolomics discover distinct metabolite profiles in children with different airway allergic diseases

**DOI:** 10.3389/fimmu.2026.1796820

**Published:** 2026-05-04

**Authors:** Yida Zhang, Peiyan Zheng, Jian-Lin Wu, Yixuan Ren, Manyun Jiang, Shiyun Li, Baoqing Sun

**Affiliations:** 1 College of Medical Technology and Engineering, Henan University of Science and Technology, Luoyang, China; 2 Department of Clinical Laboratory, State Key Laboratory of Respiratory Disease, National Center for Respiratory Medicine, National, Clinical Research Center for Respiratory Disease, Guangzhou Institute of Respiratory Health, The First Affiliated Hospital of Guangzhou Medical University, Guangzhou, China; 3 Faculty of Chinese Medicine, School of Pharmacy & State Key Laboratory of Mechanism and Quality of Chinese Medicine, Macau University of Science and Technology, Taipa, Macau SAR, China; 4 The First Clinical Medical College of Kunming Medical University, Kunming, China

**Keywords:** airway allergic disease, pediatric patients, derivatization, metabolic pathway, metabolomics

## Abstract

Allergic rhinitis (AR), asthma, and their combined allergic rhinitis and asthma syndrome (CARAS) frequently coexist. However, the underlying pathophysiological and metabolic mechanisms, as well as reliable diagnostic differentiation, remain challenging. Carboxyl-containing metabolites (CCMs) have been implicated in the pathogenesis of these conditions; therefore, this study aimed to comprehensively profile serum CCMs in pediatric patients. Sera from 63 children with AR, 41 with asthma, and 90 with CARAS, sensitized to *Dermatophagoides farina* and/or *Dermatophagoides pteronyssinus*, were analyzed using ultra-high-performance liquid chromatography-quadrupole-time-of-flight mass spectrometry coupled with 5-(diisopropylamino) amylamine derivatization. The results identified 100 differentially expressed metabolites common across them, alongside disease-specific alterations of 23, 17, and 31 unique to AR, asthma, and CARAS, respectively. These findings reveal that allergic airway diseases share a core metabolic disturbances that may reflect immune dysregulation and potentially involve gut dysbiosis, and yet exhibit distinct profiles: AR shows localized amino acid perturbations, asthma displays systemic lipid remodeling, and CARAS manifests as a distinct comorbid phenotype combining both features.

## 1 Introduction

In recent years, the prevalence of allergic airway diseases (AADs) has risen annually, largely due to increasing environmental pollution, resulting in significant global health and economic burdens (1). AADs—including allergic rhinitis (AR), asthma, and combined AR and asthma syndrome (CARAS)—are chronic inflammatory disorders of the respiratory tract (1) that affect different target organs (2). The pathologic processes that occur in the nasal mucosa of AR, lower airway of asthma, and both united airways of CARAS, reflect common manifestations of a generalized chronic disorder, including recurrent inflammation, airway hyperresponsiveness, mucus hypersecretion, and reversible airway obstruction induced by the inflammatory cellular response, which are both a local and systemic inflammatory process (3).

The pathological mechanisms of AADs are well known sharing similar inflammatory process (2). A type 2 immune response is triggered *in vivo* when individuals are exposed to airborne allergens, resulting in the involvement of multiple immune cells and the release of inflammatory mediators (4). For now, nearly 500 million and 300 million individuals worldwide suffer from AR and asthma, respectively, with a high prevalence, especially in industrialized countries (5). In addition, owing to their coexistence, up to 80% of patients with asthma are affected by AR, and approximately 40% of individuals with AR have concomitant asthma, suggesting a large number of CARAS patients in clinic (6). Despite the physiological similarities and close interrelatedness among all AADs, the specific associations and distinctions between them remain unclear. This is especially true regarding the metabolic alterations that occur during their pathological states. Therefore, there is a pressing need to delve into the pathological mechanisms underlying bronchial asthma and AR, with a special focus on CARAS.

Metabolomics is an emerging technology following genomics and proteomics, aiming to comprehensively analyze dynamic changes in a wide array of metabolites within a given sample, followed by in-depth data mining and bioinformatics analysis (7). Currently, metabolomics—leveraging advanced high-throughput analytical technologies such as mass spectrometry and nuclear magnetic resonancespectroscopy, together with bioinformatics tools—enables the systematic study of metabolic alterations in biological systems, offering high throughput, sensitivity, and specificity (8, 9). As is well known, fatty acid derivatives, particularly eicosanoids, play a key role in modulating the synthesis of inflammatory mediators (e.g., leukotrienes) and regulating the Th1/Th2 immune balance, thereby significantly influencing the pathogenesis of AADs (10). Based on metabolomics, our previous studies in patients with allergic asthma have also shown that levels of certain eicosanoids, particularly 12-hydroxyeicosatetraenoic acid (12-HETE) and 15-HETE, were significantly elevated compared to healthy, and notably decreased after subcutaneous immunotherapy (11, 12).

In light of these findings, this study employs a highly specific and sensitive derivatization approach using 5-(diisopropylamino) amylamine (DIAAA) (13), combined with ultrahigh-performance liquid chromatography quadrupole time-of-flight mass spectrometry (UHPLC-Q-TOF/MS), to profile serum carboxyl-containing metabolites (CCMs) across different types of AADs within a comprehensive metabolomics framework. The primary objective is to identify common signature metabolites across different AADs and elucidate the underlying metabolic pathways, thereby uncovering shared metabolic mechanisms. Second, characterize the distinct metabolic signatures of individual AADs, explore their interrelationships, and identify significantly altered metabolites. These findings offer novel insights into AAD pathogenesis and provide a foundation for the development of potential biomarkers.

## 2 Materials and methods

### 2.1 Study participants

This retrospective study was conducted at the First Affiliated Hospital of Guangzhou Medical University. The study period spanned from January 2021 to December 2023. Pediatric patients aged 0–12 years were consecutively recruited from the outpatient allergy clinic during this timeframe, with no cases skipped based on severity or demographics. The study protocol was approved by the Ethics Committee of the First Affiliated Hospital of Guangzhou Medical University (ES-2025-052-02), and written informed consent was obtained from subjects or legal representatives.

A total of 212 pediatric subjects were initially identified, comprising 63 AR, 90 CARAS, 41 asthma, and 18 healthy controls. Patients were all inquired with questionnaires regarding their demographics and medical history. AR diagnosis followed the ARIA guidelines (14): presence of ≥2 symptoms (sneezing, watery rhinorrhea, nasal itching, nasal congestion) persisting >1 hour/day, accompanied by ocular symptoms (itchy eyes, tearing, redness), and nasal endoscopy showing mucosal pallor and edema. Asthma diagnosis followed the GINA guidelines (15): clinical history and examination, spirometry, and FEV1 reversibility ≥12% demonstrated at least once in the previous 6 months. CARAS was defined as the simultaneous presence of both AR and asthma fulfilling ARIA and GINA criteria, respectively, with active symptoms in both airways at enrollment (16).

Furthermore, all patients had positive skin prick test (SPT) response (wheal index ≥2) and specific IgE ≥3.50 kU/L to *Dermatophagoides farina (Der f)* and/or *D. pteronyssinus (Der p)*. This threshold (RAST Class 3+) was selected to ensure clinically relevant sensitization with moderate-to-severe disease activity suitable for metabolic profiling. Exclusion criteria were as follows: acute upper respiratory tract infection; systemic or other allergic diseases; inflammatory or septic diseases; cardiovascular diseases; liver and kidney dysfunction; systemic steroid treatment; immunotherapy; and/or use of antiallergic drugs during the 1 month preceding the study. After exclusions, 194 patients with AR, CARAS, or asthma, along with 18 normal controls, were enrolled. Clinical characteristics are in **Table 1**.

**Table 1:** Demographic characteristics of children with different AADs.

Participant characteristics	AR	Asthma	CARAS	Healthy control
	(N = 63)	(N = 41)	(N = 90)	(N = 18)
Gender, male (N (%))	49, 78%	31, 76%	53, 89%	9, 50%
Age (years)	6 (5−8)	7 (5−11)	8 (7−10)	3 (2−6)
tIgE (kU/L)	289.00 (157.76−795.91)	407.47 (252.73−882.39)	470.97 (285.42−852.47)	41.32 (15.28–63.26)
*Der p* sIgE (kU/L)	43.68 (15.94−79.41)	39.74 (17.87−93.79)	45.44 (18.52−98.91)	0.02 (0.00–0.05)
SPT: *Der p* scale (SI)	4.0 (3.0−5.0)	4.0 (4.0−5.0)	4.0 (4.0−5.5)	0.0 (0.0–1.0)
*Der f* sIgE (kU/L)	42.10 (7.86−99.19)^**^	52.58 (18.23−97.80)^##^	82.26 (59.42−100.00)	0.07 (0.00–0.10)
SPT: *Der f* scale (SI)	4.0 (3.0−5.5)^*^	5.0 (4.0−5.0)^#^	5.0 (5.0−6.0)	0.0 (0.0–0.0)

### 2.2 Sample collection and preparation

Blood samples from all subjects were collected and centrifuged with 3,712g for 10 min at 4 °C. Then, the supernatant serum was aliquoted into screw-capped cryovials and stored at -80 °C for the following tests. A sample pool, prepared by mixing 5 µL of each sample, was considered as quality control (QC). Sample preparation and metabolomics approach were approached as described in **Supplementary Material** (17).

### 2.3 Detection of IgE

The serum total IgE level and *Der p/Der f* sIgE level were detected by using an ImmunoCap1000 system (Thermo Fisher Scientific Inc., California, USA). And sample with test values of total IgE > 60 kU/L, and sIgE ≥ 3.50 kU/L were used for further metabolomic analysis.

### 2.4 Derivatization method for carboxyl-containing metabolites detection

The derivatization method was used to enhance the sensitivity and separation efficiency of CCMs. Serum samples were prepared using our previously developed approach, and 5-(diisopropylamino)-amylamine (DIAAA) was selected as the derivatization reagent (13). The detailed information about derivatization procedure, standard references and other chemical reagents are shown in **Supplementary Material**.

### 2.5 UHPLC-Q-TOF-MS analysis

Nexera X2 Shimadzu UHPLC system (Shimadzu, Kyoto, Japan) was used to separate metabolites, which consisted of an autosampler, a thermostatically r(Shimadzu, Kyoto, Japan)regulated column compartment, and a binary pump with a Waters ACQUITY UPLC HSS T3 column (2.1 × 100 mm, 1.8 μm). Mass spectrometry was conducted on an AB SCIEX TripleTOF^®^ 5600 (AB Sciex, CA, USA) accurate-mass Q-TOF/MS system. For the detailed parameters of UHPLC-Q-TOF/MS, please see the **Supporting Information**.

### 2.6 Metabolomics data processing

Raw LC-MS data were acquired using Analyst TF 1.7.1 software (AB Sciex, CA, USA). For untargeted analysis, data were processed by MarkerView 1.3.1 for compound alignment with similar elution profiles, e.g., pseudomolecular ions, retention time, etc. As for the extracted ion features, only variables that appeared in more than 70% of the total samples were recorded. Targeted analysis was approached by MultiQuant 3.0.3 with an in-house established database, which contains 340 CCMs, mainly including amino acids, eicosanoids, saturated and unsaturated fatty acids, bile acids, etc. (**Supplementary Figure 1**, **Supplementary Table 1**). After being normalized by IS peak intensity, the processed data were further uploaded and imported into SIMCA-P (version 14.1, Umetrics; Umea, Sweden) for multivariate analysis. Principal component analysis (PCA) and partial least squares-discriminant analysis (PLS-DA) were constructed to find out the critical variable, which has variable importance for the projection (VIP) ≥ 1.50, fold change (FC) >1.2 or <0.8, and *p* < 0.05. Then, these metabolites were further confirmed by their exact molecular weight (MW error < 15 ppm), MS/MS information, and available biochemical databases, such as HMDB (http://www.hmdb.ca), LipidMaps (http://www.lipidmaps.org), and METLIN (https://metlin.scripps.edu). To identify potentially disrupted metabolic pathways associated with AAD, integrative network of associations was performed using MetaboAnalyst 6.0, and visualized with the MetScape 3 App in Cytoscape (18).

### 2.7 Bioinformatics and statistical analysis

Continuous variables were expressed as medians, upper and lower ranges (IQR). Statistical differences in CCM levels among groups were analyzed using non-parametric test (Kruskal–Wallis H or Mann–Whitney test), with statistical significance set at *P* < 0.05. Correlations between candidate metabolites and IgE levels in patients with airway allergies were assessed using Pearson’s correlation coefficient analysis, after adjusting for other confounding factors. Using SPSS 22.0, receiver operating characteristic (ROC) curve analysis was used to evaluate the diagnostic performance of potential biomarkers.

## 3 Results

### 3.1 Characteristics of participants

A total of 194 pediatric subjects (63 AR, 41 asthma, and 90 CARAS patients) and 15 healthy participants were enrolled in this study. The characteristics of patients and controls are shown in **Table 1**. There was no significant difference in mean age across each group (median and IQR age: 6 (5–8) for AR, 7 (5–11) for asthma, 8 (7–10) for CARAS, and 3 (2–6) for the healthy group). All patients in the disease groups were sensitized to house dust mites, with positive SPT results to *Der f* and/or *Der p.* Compared to healthy controls, patients exhibited significantly higher levels of serum total IgE and *Der p/Der f* sIgE. Notably, CARAS patients had a higher *Der f sIgE* concentrations and greater SPT sensitivity to *Der f* than those with AR or asthma alone (**Table 1**).

Values are presented as medians [25–75 interquartile range]; Specific IgE levels ≥3.50 kU/L were set as the threshold; SPT: skin prick test, SI = mean wheal diameter of allergen/mean wheal diameter of histamine.

Overall comparisons across groups (Kruskal–Wallis test for continuous variables, Chi−square test for sex): age *P* = 0.067, tIgE *P* < 0.001, *Der p* sIgE *P* < 0.001, *Der f* sIgE *P* < 0.001, SPT *Der p P* < 0.001, SPT *Der f P* < 0.001, sex *P* = 0.031.

Pairwise comparisons: *P < 0.05, **P < 0.01 (AR *vs.* CARAS); #*P* < 0.05, ##*P* < 0.01 (Asthma *vs.* CARAS).

### 3.2 Untargeted metabolomic analysis of AADs

We first applied untargeted metabolomic analysis in the discovery cohort to determine the alterations of CCMs in airway allergic patients. All serum samples were processed and analyzed using UHPLC-MS/MS with DIAAA derivatization (17). After compound alignment, 5842 peaks were discovered and recorded. Prior to multivariate statistical analysis, the missing values in this peak matrix were processed according to the “80% rule” (19). Then datasets were normalized using Pareto scaling by SIMCA-P.

**Figure 1:**
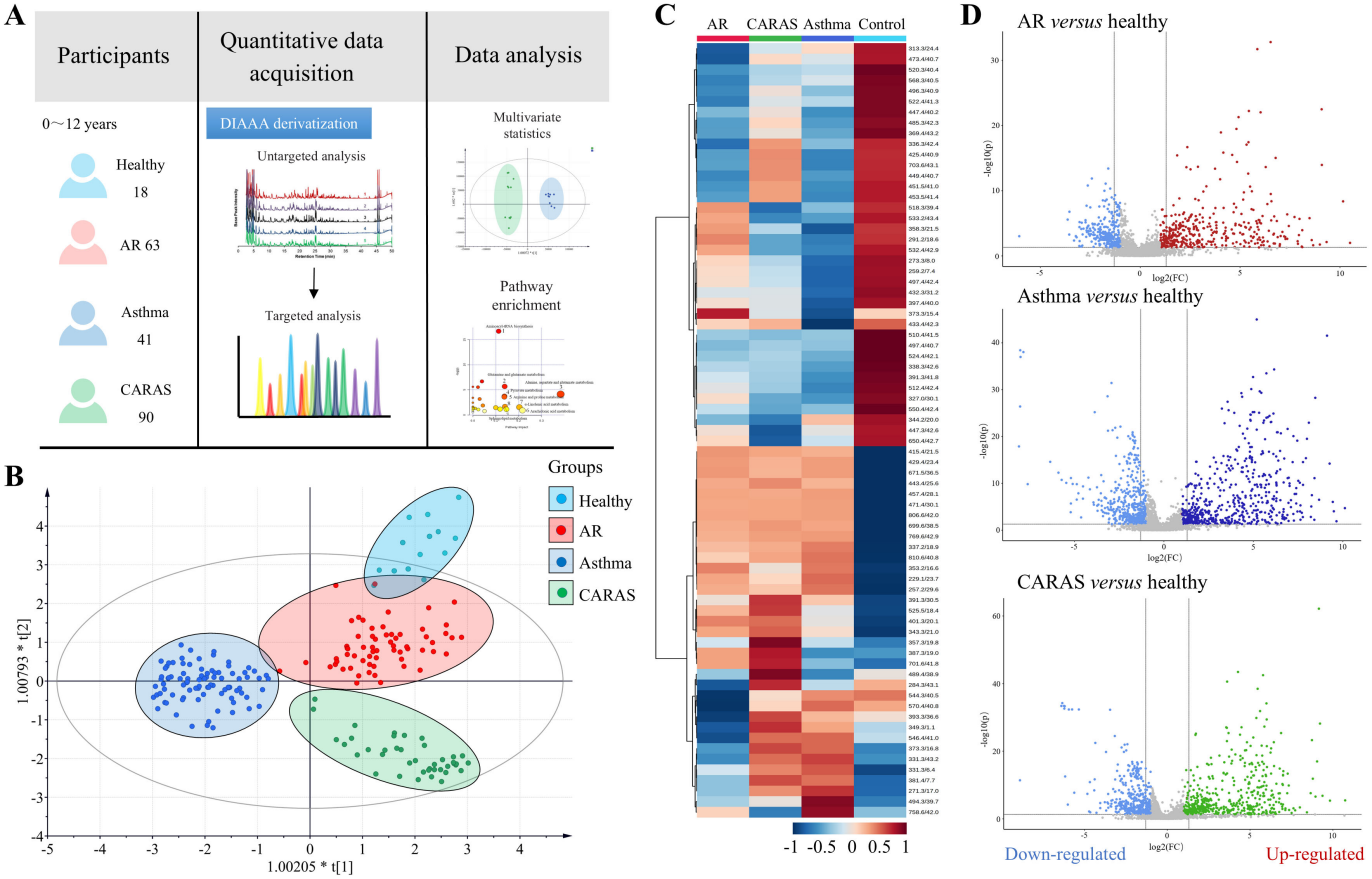
Analysis of untargeted metabolomics between patients and healthy controls. **(A)** Schematic diagram of the study design; **(B)** PLS-DA score plot of untargeted metabolomics among the four different groups; **(C)** Heatmap of 73 identified metabolites with true positive; **(D)** Volcano plots highlighted the serum metabolites that were increased (right) or decreased (left) in the AR, asthma and CARAS groups, as compared to the normal group, with log_2_ FC > 0.25 or <−0.25, and VIP score >1 in OPLS-DA.

PLS-DA was first used to visualize the data based on disease classification (R_2_Y = 0.84, Q_2_ = 0.68) (**Figure 1B**). The score plots showed a clear separation of healthy controls from patients (top area of Y-axis in **Figure 1B**, **Supplementary Figure 1**) and good separation among disease groups, AR, asthma, and CARAS. To guard against model overfitting, a default seven-fold cross-validation was also applied. Validation with 200 random permutation tests generated intercepts of R^2^ = 0.218 and Q^2^ = -0.388, indicating the PLS-DA model had good predictive ability and reliability. The relative relevance of each metabolite in the PLS-DA model was determined using the VIP value. With a VIP threshold of 1.0, 73 metabolites out of 273 peaks were further confirmed with no false positives by eliminating the adduct ions and side-products formed from the derivatization reaction (**Supplementary Table 2**). A heatmap of these 73 metabolites in four different groups was also shown in **Figure 1C**. It showed these metabolites with high (red) or low (blue) relative intensity, suggesting that distinct metabolic patterns existed among groups. These metabolites showed high (red) or low (blue) relative intensities, suggesting distinct metabolic patterns among the groups. In addition, metabolite changes between patients and healthy individuals were intuitively shown in the volcano plot (**Figure 1D**). The identification of these metabolites is described in the following section.

### 3.3 Differential metabolites characterization in untargeted metabolomic

Metabolite identification was performed according to the Metabolomics Standards Initiative (MSI) reporting guidelines. The differential metabolites were annotated using accurate mass measurement (<5 ppm), MS/MS fragmentation pattern matching against databases (Lipid Maps, METLIN, HMDB), and retention time comparison with authentic standards. According to our previous report about DIAAA-derivatization (17), metabolites were first checked for DIAAA-derived characteristic fragment ions (m/z 128, 86, [M+H-42]^+^, [M+H-84]^+^, and/or [M+H-101]^+^). Metabolites with these special MS/MS ions were considered as CCMs. For example, metabolite ID 10684 (**Supplementary Table 2**) showed a quasi-molecular ion at m/z 473.4477 (C_31_H_56_N_2_O) and product ions at m/z 431.4005, 389.3535, 372.3285, 128.1449, and 86.0978 (**Supplementary Figure 4A**); its retention time and MS/MS spectrum matched those of authentic arachidonic acid, allowing unambiguous identification (MSI Level 1). Similarly, compound 08322 (**Supplementary Table 2**, **Supplementary Figure 4B**) was identified as palmitic acid (MSI Level 1). For non-derivatized metabolites, no DIAAA-related fragment ions were observed. Instead, annotation relied on database MS/MS matching. Compound 12869 (**Supplementary Table 2**, **Supplementary Figure 4C**) exhibited MS/MS ions at m/z 104.1062 and 184.0726 (characteristic of the phosphatidylcholine head group), together with [M-H-H_2_O]^+^ at m/z 506.3585 and [M+HC_5_H_13_NO_4_P]^+^ at m/z 341.3042, leading to its annotation as LysoPC(18:0/0:0) with MSI Level 2. In total, among the 64 differential metabolites, 28 were assigned MSI Level 1 and 36 were assigned MSI Level 2 (**Supplementary Table 2**).

### 3.4 Altered targeted metabolites with different AADs

Untargeted metabolomics was performed to identify differential metabolites, followed by targeted analysis to expand metabolite coverage. With the in-house established database, we performed the targeted metabolomics analysis of 340 CCMs, mainly including amino acids, eicosanoids, saturated and unsaturated fatty acids, bile acids, etc. (**Supplementary Table 1**). These metabolites are involved in fatty acid metabolism, nitrogen metabolism, the TCA cycle, amino acid metabolism, and bile acid metabolism.

**Figure 2:**
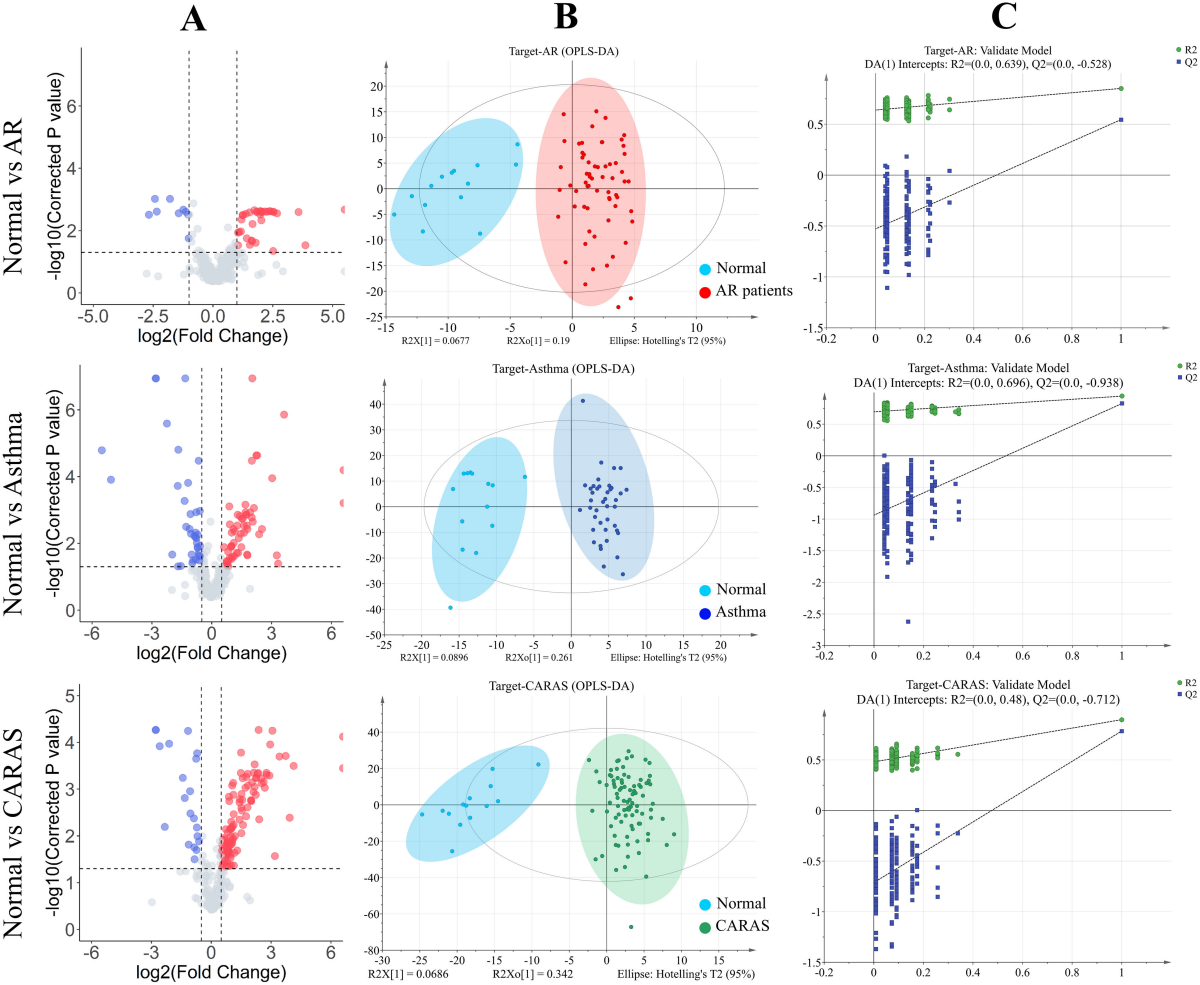
Analysis of targeted metabolomics between AAD patients and healthy controls. **(A)**Volcano plots highlighted differential metabolites of subjects in AR, asthma, and CARAS groups, compared to healthy controls. **(B)** OPLS-DA score plots of AR, asthma, and CARAS patients, compared to healthy controls. **(C)** 100 permutation tests to evaluate the quality of the OPLS-DA model.

Then, we especially focused on the differences in these target metabolites between patients with distinct AADs and healthy individuals. The supervised OPLS-DA mode was adopted with a significance threshold of 0.5 for Q^2^ and R^2^. Clear separations were found for the following groups: AR *versus* normal, cumulative R^2^Y=0.852 and Q^2^Y=0.545; asthma *versus* normal, cumulative R^2^Y=0.943 and Q^2^Y=0.826; CARAS *versus* normal, cumulative R^2^Y=0.898 and Q^2^Y=0.785 (**Figure 2**). The VIP values of each metabolite for all discrimination projections were also recorded (**Supplementary Table 3**). Candidates (VIP >1) were further validated by *P* values of Student’s unpaired *t*-test or Mann-Whitney *U* test and FC values. Compared to the normal group, the results showed that there were 17 significantly increased metabolites in the asthma group and 16 decreased, while 28 increased metabolites and 6 decreased in the CARAS group; and the number was greater in the AR group, with 73 increased and 21 decreased (**Supplementary Table 4**; *p* < 0.05, log_2_FC >0.25 or <−0.25, VIP >1). From the results, most changed metabolites influenced by disease were up-regulated, and belonged to hydroxy fatty acids and peptides. Meanwhile, lactic acid generally declined in all three disease groups, while Dihomo-γ-linolenic acid was merely decreased in AR and asthma (**Supplementary Table 4**, **Supplementary Figure 5**).

### 3.5 Metabolic pathway and enrichment analysis of AADs

**Figure 3:**
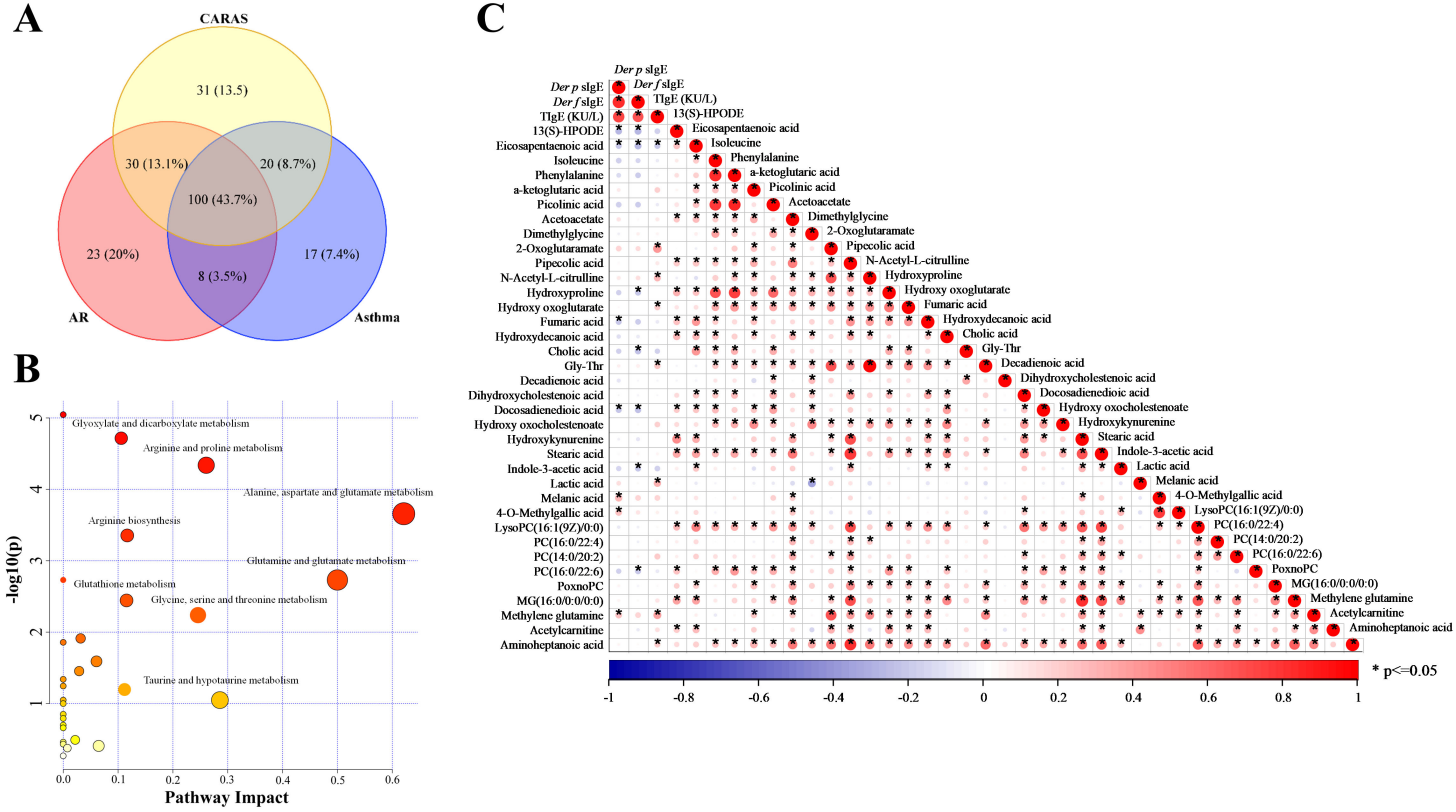
Commonly altered metabolites in AADs. **(A)** Venn diagram of differential metabolites in three different diseases. **(B)** Disturbed pathways of common metabolites in response to airway allergy. **(C)** Heat map of Pearson correlation analysis between representative metabolites and IgE levels "*" means p<0.05.

Results from both approaches were integrated by combining differential metabolite lists and removing duplicates. Venn diagram analysis showed that 162, 146, and 182 metabolites were altered in AR, asthma, and CARAS, respectively (**Figure 3A**). Among these, 100 metabolites were common to all three diseases, further suggesting that these three AADs may have similar mechanisms under pathological conditions. MetaboAnalyst 6.0 was then used to visualize the metabolic pathways affected by AADs. Based on these 100 commonly differentially expressed metabolites, the results showed that AADs mainly interfere with alanine, aspartate and glutamate metabolism, glutamine and glutamate metabolism, taurine and hypotaurine metabolism, arginine and proline metabolism, glycine, serine and threonine metabolism, glyoxylate and dicarboxylate metabolism, arginine biosynthesis, glutathione metabolism (**Figure 3B**).

The pathogenesis of AADs is mainly due to the production of corresponding IgE antibodies in the body when allergens enter atopic individuals. IgE level play an important role in the entire process, affecting the prognosis of AADs. Therefore, correlations between metabolites and IgE levels in patients were investigated, and *P* < 0.05 was considered relevant (**Supplementary Table 4**). As a result, we found 36 metabolites that were significantly correlated with the levels of tIgE, *Der p* sIgE or *Der f* sIgE. Among them, 13(S)-HPODE was negatively correlated with both *Der p* and *Der f* sIgE, whereas pipecolic acid, melanic acid, 4-O-methylgallic acid, PC(16:0/22:4), PC(14:0/20:2), and methylene glutamine were positively correlated with *Der p* sIgE and tIgE.

### 3.6 Distinct metabolic profiles and pathways of different AADs

**Figure 4:**
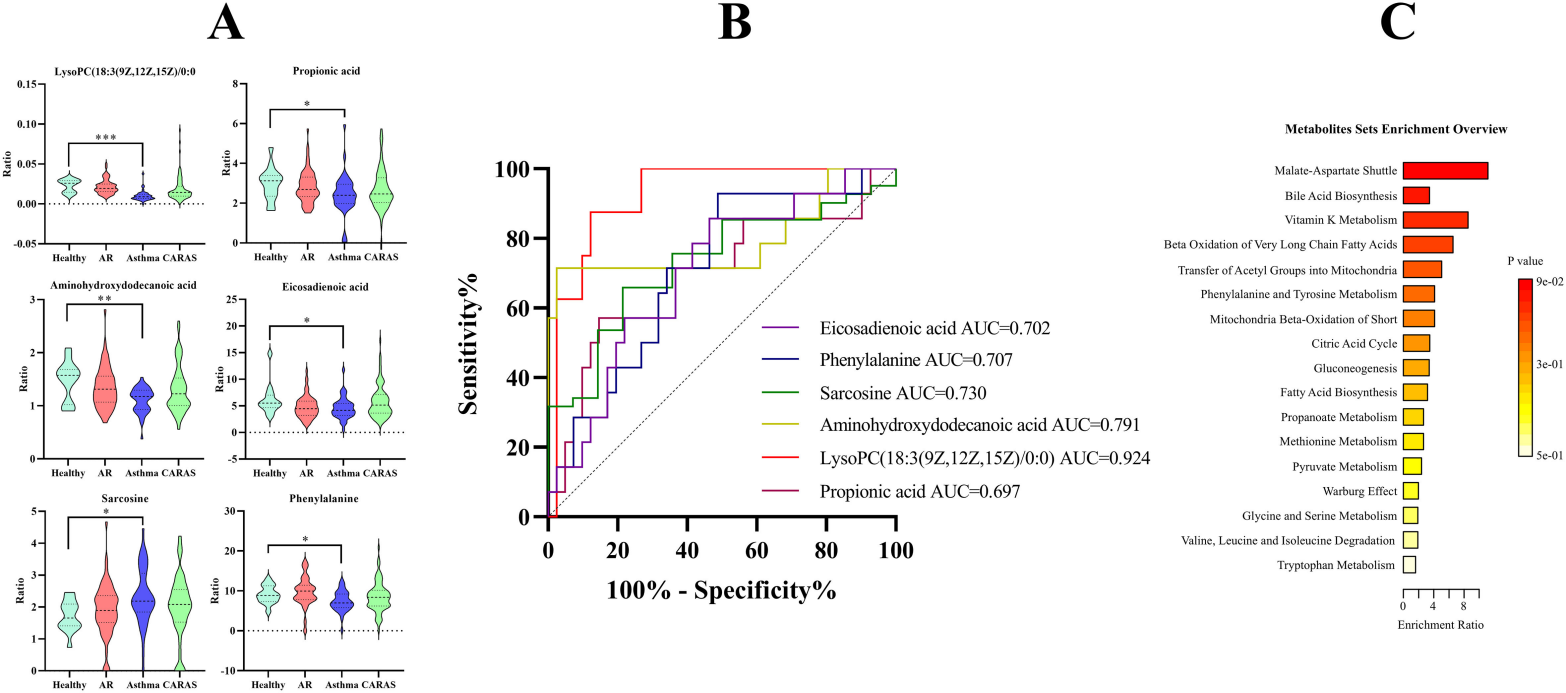
Specifically altered metabolites in patients with allergic asthma. **(A)** Box plots of candidate biomarkers. *, **, *** denoted *P* < 0.05, *P* < 0.01, and *P* < 0.001, compared to healthy controls. **(B)** ROC curve of significantly changed candidate biomarkers. **(C)** Metabolic pathway analysis based on differentially expressed metabolites.

Venn diagram suggested 23, 17 and 31 specific metabolites that were significantly altered only in AR, asthmatic, and CARAS, respectively (**Figure 3A**). To clarify these specific metabolites of different AADs, the candidate metabolites were diagnosed using ROC curve analysis to preliminarily evaluate their diagnostic efficacy (**Supplementary Table 5**). Among these differential metabolites, there were 6 candidate biomarkers found in asthma (**Figure 4A**), which has good diagnostic performance, namely, lysoPC(18:3(9Z,12Z,15Z)/0:0), sarcosine, phenylalanine, eicosadienoic acid, propionic acid, docosatrienoic acid. Except for sarcosine, rest of them were decreased in patients with asthma. And, areas under the ROC curve (AUCs) of them were 0.924, 0.730, 0.707, 0.702, 0.697, and 0.679, respectively (**Figure 4B**). Similarly, four and eight candidate biomarkers were also found in AR and CARAS, respectively. However, unlike asthma, most of these metabolites were upregulated in CARAS (**Supplementary Figure 7**). These potential biomarkers can be used to discriminate among different AAD, and all remained significant after age adjustment (**Supplementary Table 6**).

To delve deeper into the metabolic pathways that may be more susceptible to these distinct AADs, unique metabolites associated with each disease were utilized for pathway analysis. As shown in **Figure 4C**, the most disease-associated pathways related to asthma included malate-aspartate shuttle, vitamin K metabolism, transfer of acetyl groups into mitochondria, phenylalanine and tyrosine metabolism, mitochondrial beta-oxidation of short-chain saturated fatty acids, citric acid cycle, etc. In contrast, distinct metabolites of AR implicated in a diverse array of other metabolic pathways, such as aspartate metabolism, arginine and proline metabolism, glycine and serine metabolism, valine, leucine and isoleucine degradation, urea cycle, fatty acid biosynthesis, propanoate metabolism. CARAS was associated with fatty acid biosynthesis, bile acid biosynthesis, beta-oxidation of very long-chain fatty acids (LCFAs), glycerolipid metabolism, fatty acid elongation in mitochondria, fatty acid metabolism, steroid biosynthesis (**Supplementary Figure 7**). The differential distinct metabolites were related to distinct underlying mechanisms, and have potential to aid in elucidating the pathogenesis of different AADs.

## 4 Discussion

AADs are highly prevalent and significantly impair patients’ quality of life. More concerning, they affect over 30% of children (20). AADs involve different target organs (airways *vs.* nasal cavity) and have distinct inflammatory pathways. Therefore, relying solely on clinical symptoms and routine examinations can sometimes be difficult to accurately distinguish specific disease types, potentially leading to misdiagnosis or delayed treatment. However, current understanding of diagnostic classification, pathophysiological mechanisms, and particularly the metabolic pathway alterations in different AADs, remains limited. Moreover, there is still a lack of early, specific biomarkers for precise AAD discrimination.

Given that *Der p* and *Der f* are the dominant allergens in Guangdong, China (21), we systematically recruited pediatric patients with AADs specifically sensitized to *Der p/Der f* from our hospital cohort. Serum *Der f* sIgE was selectively higher in CARAS than in children with AR or asthma (*P* < 0.01), whereas *Der p* sIgE did not differ among them. This species-selective pattern suggests that Th2-biased inflammation characteristic of CARAS is amplified not by global mite-allergen load, but by immune recognition of proteins uniquely or disproportionately expressed by *Der f* (22).

CCMs have been closely implicated in the pathogenesis of AADs. However, their quantification remains challenging because of ultra-low endogenous levels, wide polarity ranges, and severe matrix interference (23). To overcome these limitations, we applied a sensitive workflow that integrates DIAAA derivatization with UHPLC-MS/MS and accommodates both targeted and untargeted metabolomics. Derivatization lowered the limits of detection (LODs) to the femtogram range—two orders of magnitude better than underivatized method. By curating an in-house library of 340 CCMs, we achieved fg-level quantification of critical mediators, including HETEs, prostaglandin D2, thromboxane B2 and leukotriene B4 (24). To our knowledge, this is the first metabolomics study exploring the metabolic pathways, providing insights into the clinical diagnosis and subtyping of AAD.

Metabolomics bridges molecular and clinical phenotypes, offering a powerful tool to decipher pathogenesis and refine diagnosis and treatment of pediatric AADs. In this study, we integrated targeted and untargeted approaches: untargeted metabolomics provided a comprehensive snapshot of the sample’s intrinsic metabolic profile, while targeted focused on predefined metabolites of interest, including low−abundance species (25). By combining the differential metabolites from both approaches after duplicate removal, we yielded 385 confidently annotated metabolites, primarily classified as fatty acids, peptides, organic acids, amino acids, bile acids, TCA intermediates, and lipids (**Supplementary Table 3**). Among them, 162, 146 and 182 metabolites were significantly altered in AR, asthma and CARAS, respectively.

**Figure 5:**
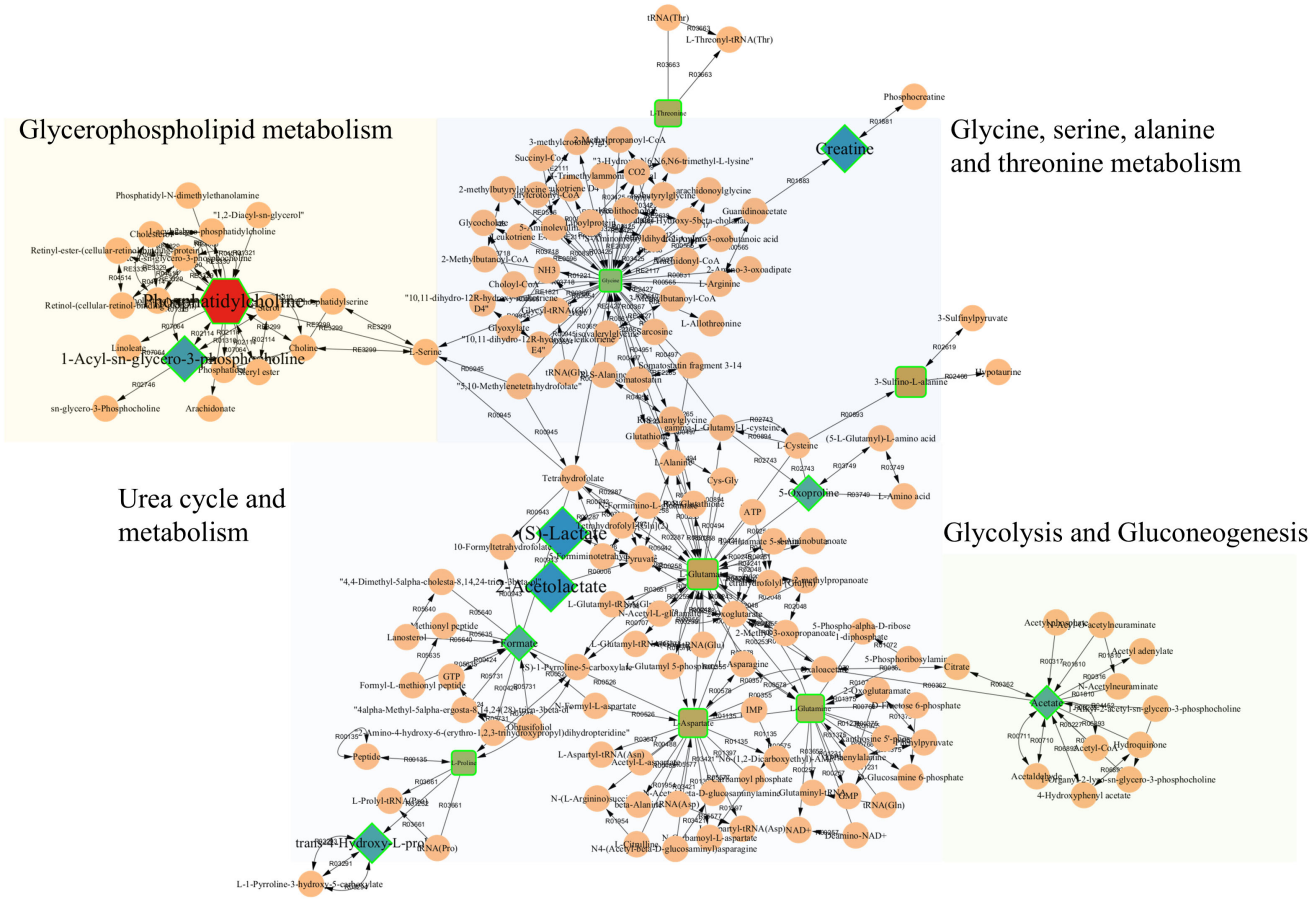
Perturbed metabolic regulatory network in response to all AADs. Nodes with distinct color and shape represent key metabolites: Intensity of color reflects the statistical significance (P-value), while node size is proportional to the magnitude of fold change.

Among these changed metabolites, 100 were consistently dysregulated across all three diseases. These perturbed metabolites were significantly enriched in several key metabolic pathways, including alanine, aspartate, and glutamate metabolism; glutamine and glutamate metabolism; taurine and hypotaurine metabolism; glycine, serine, and threonine metabolism; glycerophospholipid metabolism; urea cycle and other associated metabolic processes (**Figure 3B**). Notably, these pathways exhibited extensive interconnectivity, collectively forming an integrated metabolic network within the organism. To further elucidate the underlying pathophysiological mechanisms, a correlation network of potential biomarkers associated with AAD was constructed, as illustrated in **Figure 5**.

Metabolomic analysis revealed a set of shared metabolic disturbances in AADs patients, consistent with an interconnected pathophysiological network. The consistent decrease in short-chain fatty acids (SCFAs), such as acetic acid and hexanoic acid (**Supplementary Table 3**), across disease groups may be linked to gut microbiota alterations (26, 27), though this interpretation remains correlative and hypothesis-generating in the absence of microbiome sequencing. Concurrently, increased levels of the pro-inflammatory prostaglandin PGF_1_ α, decreased of anti-inflammatory mediator docosahexaenoic acid (DHA), and elevated levels of membrane phospholipids, like PC(16:0/22:6) containing arachidonic and DHA precursors, collectively reflect a shift in lipid metabolism toward a pro-inflammatory and pro-oxidative state, alongside a probable imbalance in the synthesis of specialized pro-resolving mediators (28).

Lipids are not only the main components of pulmonary surfactant, which can maintain lung function by reducing the surface tension of alveolar gas-liquid interface, but also play an important regulatory role in inflammatory response and oxidative stress (29). Thus, AADs are always with lipid metabolism disorder. Studies have shown that PC metabolite levels were dysregulated in patients with AAD, such as Lysophosphatidylglycerol (LPG) 18:0 (30); PC (22∶1/20∶5), PC (18∶0/22∶3), PC (20∶4/22∶5), PC (18∶0/22∶5) (31). In present study, PC(16:0/22:4(7Z,10Z,13Z,16Z)) and PC(16:0/22:6(4Z,7Z,10Z,13Z,16Z,19Z)) significantly upregulated in all AADs, whereas LysoPC(18:0/0:0) and LysoPC(20:1(11Z)/0:0) downregulated. This reveals that with AAD conditions, phospholipid remodeling of plasma membrane was activated, concomitant with relative alterations in sPLA_2_ activity, ultimately culminating in a pro-inflammatory milieu and cellular dysfunction (32). Due to the enhanced sensitivity of derivation, other CCMs, such as undecanoic acid, tetradecanedioic acid, suberic acid, and nonanedioic acid were found to be markedly elevated in AADs, suggesting increased mobilization and oxidation of saturated and medium-chain fatty acids. This metabolic profile may reflect enhanced utilization of fatty acids as energy substrates by activated immune cells, heightened oxidative stress driving ω-oxidation and subsequent dicarboxylic acid formation, or a specific remodeling of mitochondrial β-oxidation pathways during inflammation (33, 34).

Nevertheless, metabolomic profiling revealed distinct metabolic signatures about different AAD, likely reflecting variations in disease localization and severity. Isolated AR was characterized primarily by marked activation of local upper airway tryptophan-kynurenine metabolism (e.g., elevated aminophenyl-dioxobutanoic acid, hydroxy-kynurenine) and specific changes in branched-chain amino acids (35). In contrast, lower airway inflammation in asthma was associated with more prominent disturbances in lysophosphatidylcholine metabolism (e.g., LysoPC(18:3(9Z,12Z,15Z)/0:0)), alterations in polyunsaturated fatty acid profiles (e.g., eicosadienoic acid), and abnormalities in primary bile acids (e.g., cholic acid). This implies more active membrane phospholipid degradation, involvement of lipid mediators, and potential gut-liver-lung axis interactions in asthma (36, 37).

Notably, the metabolic signature of CARAS did not represent a simple superposition of AR and asthma, but rather a distinct comorbidity with its own metabolic features. Its features included a combination of partial metabolic alterations observed in both upper and lower airways, coupled with a pronounced systemic metabolic dysregulation. Key evidence included significant perturbations in SCFAs (e.g., isovaleric acid, valeric acid) and microbiota-derived tryptophan metabolites (e.g., indole-3-acetic acid), findings that are consistent with the hypothesis of a disrupted microbiota--immune crosstalk (38). Concurrently, extensive alterations were observed in LCFA profiles, sterol metabolites (e.g., cholestenoic acid, ursodeoxycholic acid), and phospholipid metabolites (e.g., some LysoPCs), collectively indicating comprehensive remodeling of lipid metabolism and impaired cellular membrane stability (39).

These findings suggest that along the disease continum from AR to CARAS and asthma, metabolic disturbances evolve from a relatively local to a systemic scale, with influence of lipid metabolism and microbiome becoming increasingly prominent. Therefore, characteristic metabolites such as specific lysophospholipids, microbiota-related SCFAs, and bile acids not only provide potential biomarkers for differentiating these clinical phenotypes but also offer novel metabolic perspectives for investigating their comorbid mechanisms (e.g., in CARAS) and disease progression trajectories.

## 5 Conclusion

In summary, this study represents the first application of a derivatization-based metabolomic approach in a pediatric cohort, delineating both shared and distinct metabolic profiles underlying AADs: AR, asthma, and CARAS. Common metabolic disturbances in AADs reflect an interconnected pathophysiological network may involve gut dysbiosis, along with impaired epithelial barrier integrity, and mucosal immune dysregulation, highlighting the key mechanisms of inflammation and metabolic imbalance. While AR was characterized by localized perturbations in upper airway amino acid metabolism; asthma by systemic lipid remodeling involving lysophospholipids and bile acids; and CARAS by a distinct dysregulation that integrates features of both, prominently featuring microbial-derived metabolites and extensive disruptions across lipid metabolic networks. These results provide a more detailed understanding for AADs, and nominate specific metabolite panels as potential biomarkers, offering a novel metabolic framework for understanding the metabolic relationships among localized allergic inflammation and systemic comorbid disease in children.

## Data Availability

The original contributions presented in the study are included in the article/**Supplementary Material**. Further inquiries can be directed to the corresponding author.

## Glossary

AAD: airway allergic disease
CCM: carboxyl-containing metabolite
AR: allergic rhinitis
CARAS: combined allergic rhinitis and asthma syndrome
*Der p*: *Dermatophagoides pteronyssinus*
*Der f*: *Dermatophagoides farina*
IgE: Immunoglobulin E
FA: fatty acid
DIAAA: 5-(diisopropylamino) amylamine
OPLS-DA: orthogonal projections to latent structures discriminant analysis
VIP: variable importance in projection
UHPLC-Q-TOF/MS: ultrahigh performance liquid chromatography-quadrupole-time of flight mass spectrometry.
