# Correction: Metabolomics discover distinct metabolite profiles in children with different airway allergic diseases

**DOI:** 10.3389/fimmu.2026.1933865

**Published:** 2026-07-23

**Authors:** Yida Zhang, Peiyan Zheng, Jian-Lin Wu, Yixuan Ren, Manyun Jiang, Shiyun Li, Baoqing Sun

**Affiliations:** 1 College of Medical Technology and Engineering, Henan University of Science and Technology, Luoyang, China; 2 Department of Clinical Laboratory, State Key Laboratory of Respiratory Disease, National Center for Respiratory Medicine, National, Clinical Research Center for Respiratory Disease, Guangzhou Institute of Respiratory Health, The First Affiliated Hospital of Guangzhou Medical University, Guangzhou, China; 3 Faculty of Chinese Medicine, School of Pharmacy & State Key Laboratory of Mechanism and Quality of Chinese Medicine, Macau University of Science and Technology, Taipa, Macau SAR, China; 4 The First Clinical Medical College of Kunming Medical University, Kunming, China

**Keywords:** airway allergic disease, pediatric patients, derivatization, metabolic pathway, metabolomics

The affiliations for author Peiyan Zheng were erroneously written as “2, 3”. The correct affiliation numbering should be: “3, 2”.

Authors Yida Zhang and Peiyan Zheng were erroneously omitted as equal contributing authors. The original version of this article has been updated.

