# Supplementary material for: Metabolomics discover distinct metabolite profiles in children with different airway allergic diseases
Source: Front Immunol. 2026 May 4;17:1796820. doi: 10.3389/fimmu.2026.1796820 (PMC13180947; doi:10.3389/fimmu.2026.1796820)
Supplement: Supplementary file 1 [file DataSheet1.pdf]

Supplementary Materials for

Metabolomics Discover Distinct Metabolite Profiles in Children with Different Airway Allergic Diseases

Yida Zhang[a, b], Peiyan Zheng[b,c], Jian-Lin Wu[c], Yixuan Ren[d], Manyun Jiang[b], Shiyun Li[b], Baoqing Sun[b*]

[a] College of Medical Technology and Engineering, Henan University of Science and Technology, Luoyang 471023, China

[b] Department of Clinical Laboratory, State Key Laboratory of Respiratory Disease, National Center for Respiratory Medicine, National Clinical Research Center for Respiratory Disease, Guangzhou Institute of Respiratory Health, The First Affiliated Hospital of Guangzhou Medical University, Guangzhou 510120, China; Guangzhou National Laboratory, Guangzhou 510005, China; Guangdong Provincial Clinical Research Center for Laboratory Medicine, Guangzhou 510080, China

[c] Faculty of Chinese Medicine, School of Pharmacy & State Key Laboratory of Mechanism and Quality of Chinese Medicine, Macau University of Science and Technology, Avenida Wai Long, Taipa, Macau, 999078, China.

[d] The First Clinical Medical College of Kunming Medical University, Kunming 650500, China

*Correspondence:

Baoqing Sun, The First Affiliated Hospital of Guangzhou Medical University

sunbaoqing@vip.163.com

Supplementary Materials includes:

Figure S1 | Structures of representative carboxyl-containing metabolites in serum.

Figure S2 | Untargeted metabolomic analyses were performed using sera obtained from the discovery cohort. **(A)** OPLS-DA score plots for AR, CARAS and asthma group, compared to normal group. **(B)** 100 permutation tests to evaluate the quality of the OPLS-DA model.

Figure S3 | Comparison of representative metabolites (lactic acid and dihomo-γ-linolenic acid) in children with AADs.

Figure S4 | Heatmap of 73 metabolites found in four different groups by untargeted metabolomics.

Figure S5 | MS/MS spectra of DIAAA-derivatized metabolites: arachidonic acid **(A)**, palmitic acid **(B)**, and non-derivatized metabolite: LysoPC(18:0/0:0) **(C)**.

Figure S6 | Pathway enrichment of the significant metabolites identified by untargeted and targeted approaches using the metabolic datasets. (**A**) Barchart view, (**B**) network view.

FIGURE S7 | Comparison of representative biomarker candidates specifically found in children with AR (**A**) and CARAS (**B**). *, **, *** denoted *P* <0.05, *P* <0.01, and *P* <0.001 in patients compared to healthy control. Metabolic pathway analysis based on differentially expressed metabolites in the AR (**C**) and CARAS (**D**).

TABLE S1 | Derivatized standards used in targeted standards analysis.

TABLE S2 | Identified differential metabolites in the serum of AADs patients by untargeted metabolomics.

TABLE S3 | Total differential metabolites between AAD patients and healthy controls by targeted and untargeted metabolomics.

TABLE S4 | Pearson's correlation analysis between altered metabolites and IgE levels.

TABLE S5 | Diagnostic performance of specific metabolites for each AADs

1. Chemicals and materials

MS-grade acetonitrile and methanol were purchased from Anaqua Chemicals Supply (Houston, TX, United States) and MS-grade formic acid was provided by Sigma-Aldrich Laboratories, Inc., Deionized water was supplied by a Millipore water purification system (Millipore, United States). DIAAA, O-(7-azabenzotriazol-1-yl)-N,N,N',N'-tetramethyluronium hexafluorophosphate (HATU), 1-hydroxybenzotriazole hydrate (HOBt), triethylamine (TEA), and dimethyl sulfoxide (DMSO, MS grade) were also bought from Sigma-Aldrich Laboratories, Inc.

Carboxyl-containing metabolites standards, such as amino acid mixtures, TCA cycle intermediates, and short-chain fatty acids (SCFAs) were purchased from Sigma-Aldrich Laboratories, Inc., (St. Louis, MO, USA). Fatty acid standards, including 5(S)-, 8(S)-, 11(S)-, 12(S)-, and 15(S)-HETEs, 5(S)-, 12(S)-, and 15(S)-HPETEs, 9(S)- and 13(S)-HPODEs, 5(S)-, 12(S)-, and 15(S)- hydroxyeicosapentaenoic acids (HEPEs), 13(S)-hydroxyoctadecatrienoic acid (HOTrE); Prostaglandin A2 (PGA2), PGB2, PGD2, PGE2, PGJ2, PGE1, PGF1α, TXB2, 12(S)-hydroxyheptadecatrienoic acid (HHTrE), and 13,14-dihydro-15-keto PGF2α, were purchased from Cayman Chemical (Ann Arbor, MI, USA). The internal standards, isotope-labeled 2(S)-HETE-d8 and PGD2-d4 were provided by Cayman Chemical (Ann Arbor, MI, USA), while 4-Cl-phenylalanine was obtained from Sigma-Aldrich Laboratories, Inc. (St. Louis, MO, USA). Other standards, such as hexanoic acid, octanoic acid, decanoic acid, dodecanoic acid, myristic acid, stearic acid, heptadecanoic acid, docosahexaenoic acid, eicosapentaenoic acid, docosapentaenoic acid, oleic acid, chenodeoxycholic acid, deoxycholic acid, and isodeoxycholic acid were obtained from Sigma-Aldrich (St. Louis, MO, USA) and J&K Scientific (Beijing, China).

2. Methods

2.1 Metabolite extraction

To extract metabolites from serum samples, 200 μL cold methanol was added to 50 μL of each serum, and then thoroughly vortexed. After that, the mixture was centrifuged at 13,000 x *g* for 10 minutes at 4 °C, and repeated the above extraction for

three times. Then the combined supernatants were dried in a vacuum centrifuge at 4°C. The residue was stored at −20 °C before derivatization.

2.2 DIAAA Derivatization

The derivatization was performed according to our previous method with little revision [1]. Briefly, HOBt and HATU were separately dissolved in DMSO at the concentration of 20 mM, while the DIAAA-TEA solution was prepared by dissolving 100 mmol of DIAAA and 200 mmol of TEA in 1 mL of DMSO. Thereafter, the dried residue of sample was sequentially mixed with 5 μL of HOBt, 5 μL of DIAAA-TEA solution, and 5 μL of HATU, followed by 1 min incubation at room temperature. Finally, 35 μL of acetonitrile was added to make up to the final volume of 50 μL, and 1 μL of the supernatant was directly injected into UHPLC-Q-TOF/MS. The samples were injected in random order and a QC sample was injected every 7 samples.

2.3 UHPLC-Q-TOF/MS analysis

Nexera X2 Shimadzu UHPLC system (Shimadzu Corporation, Kyoto, Japan) and binary pump with Waters ACQUITY UPLC HSS T3 column (2.1 × 100 mm, 1.8 μm) were employed for the separation of all metabolites. The column temperature was maintained at 35 °C and the autosampler was set at 4 °C. Mobile phase A and B were 0.1% formic acid-containing water and 0.1% formic acid-containing acetonitrile, respectively, and the gradient was set as follows: 0-0.5 min, 2% to 5% B; 0.5-2.5 min, 5%-6% B; 2.5-4.5 min, 6%-7% B; 4.5-5.5 min, 7%-7.3% B; 5.5-7.5 min, 7.3%-7.8% B; 7.5-11 min, 7.8%-9% B; 11-13 min, 9%-14% B; 13-18 min, 14%-23% B; 18-19 min,; 23%-25% B; 19-26 min, 25%-33% B; 26-26.5 min, 33%-35%; 26.5-34.5 min, 35%-47% B; 34.5-38 min, 47%-60% B; 38-40 min, 60%-95% B; 39-43.9 min, 95%; 44 min, 2% B. The injection volume was 1 μL, and the flow rate was 0.3 mL min^{-1}.

Mass spectrometry was conducted by AB SCIEX TripleTOF® 5600 (AB Sciex, CA, USA) accurate-mass Q-TOF/MS system. The data was collected using information-dependent acquisition (IDA) mode with mass range m/z 100–1200 for TOF-MS scan, and m/z 50–1200 for TOF-MS/MS scan. After optimization with the standards, the instrument was operated in positive full scan mode with the following MS parameters: nebulizer gas, 50 L/min; heater gas, 50 L/min; curtain gas, 35 L/min;

ion spray voltage, 5500 V; declustering potential, 80 V; collision energy spread, ±15 eV. The experiments were run with 150 ms accumulation time for TOF-MS and 50 ms accumulation time for TOF-MS/MS. Meanwhile, the accurate MS and MS/MS measurements were acquired with the Automated Calibration Delivery System (reference masses: m/z 322.0481, 622.0289, 922.0098).

TABLE S1 | Derivatized standards used in targeted standards analysis

Classification	Name	Formula	Derivatization formula	$[M+H]^+$
	Arachidonoic acid	$C_{20}H_{32}O_2$	$C_{31}H_{56}N_2O$	473.4465
	LTB4	$C_{20}H_{32}O_4$	$C_{31}H_{56}N_2O_3$	505.4364
	12(S)-HPETE	$C_{20}H_{32}O_4$	$C_{31}H_{56}N_2O_3$	505.4364
	15(S)-HPETE	$C_{20}H_{32}O_4$	$C_{31}H_{56}N_2O_3$	505.4364
	5(S)-HPETE	$C_{20}H_{32}O_4$	$C_{31}H_{56}N_2O_3$	505.4364
	13(S)-HPODE	$C_{18}H_{32}O_4$	$C_{29}H_{56}N_2O_3$	481.4364
	9(S)-HPODE	$C_{18}H_{32}O_4$	$C_{29}H_{56}N_2O_3$	481.4364
	15(S)-HETE	$C_{20}H_{32}O_3$	$C_{31}H_{56}N_2O_2$	489.4415
	11(S)-HETE	$C_{20}H_{32}O_3$	$C_{31}H_{56}N_2O_2$	489.4415
	12(S)-HETE	$C_{20}H_{32}O_3$	$C_{31}H_{56}N_2O_2$	489.4415
	8(S)-HETE	$C_{20}H_{32}O_3$	$C_{31}H_{56}N_2O_2$	489.4415
	5(S)-HETE	$C_{20}H_{32}O_3$	$C_{31}H_{56}N_2O_2$	489.4415
	15(S)-HEPE	$C_{20}H_{30}O_3$	$C_{31}H_{54}N_2O_2$	487.4258
PUFAs	12(S)-HEPE	$C_{20}H_{30}O_3$	$C_{31}H_{54}N_2O_2$	487.4258
	5(S)-HEPE	$C_{20}H_{30}O_3$	$C_{31}H_{54}N_2O_2$	487.4258
	13(S)-HOTrE	$C_{18}H_{30}O_3$	$C_{29}H_{54}N_2O_2$	463.4258
	15(S)-HETrE	$C_{20}H_{34}O_3$	$C_{31}H_{58}N_2O_2$	491.4571
	PGA2	$C_{20}H_{30}O_4$	$C_{31}H_{54}N_2O_3$	503.4207
	PGB2	$C_{20}H_{30}O_4$	$C_{31}H_{54}N_2O_3$	503.4207
	PGJ2	$C_{20}H_{30}O_4$	$C_{31}H_{54}N_2O_3$	503.4207
	PGE2	$C_{20}H_{32}O_5$	$C_{31}H_{56}N_2O_4$	521.4313
	PGD2	$C_{20}H_{32}O_5$	$C_{31}H_{56}N_2O_4$	521.4313
	13, 14-dehydro-15-keto PGD2	$C_{20}H_{32}O_5$	$C_{31}H_{56}N_2O_4$	521.4313
	13, 14-dehydro-15-keto PGE2	$C_{20}H_{32}O_5$	$C_{31}H_{56}N_2O_4$	521.4313

	13, 14-dehydro-15-keto PGF2α	$C_{20}H_{34}O_5$	$C_{31}H_{58}N_2O_4$	523.4469
	11β-PGF2α	$C_{20}H_{34}O_5$	$C_{31}H_{58}N_2O_4$	523.4469
	PGF2β	$C_{20}H_{34}O_5$	$C_{31}H_{58}N_2O_4$	523.4469
	PGF2α	$C_{20}H_{34}O_5$	$C_{31}H_{58}N_2O_4$	523.4469
	PGE1	$C_{20}H_{34}O_5$	$C_{31}H_{58}N_2O_4$	523.4469
	PGF1α	$C_{20}H_{36}O_5$	$C_{31}H_{60}N_2O_4$	525.4626
	TXB2	$C_{20}H_{34}O_6$	$C_{31}H_{58}N_2O_5$	539.4418
	6-keto-PGF1α	$C_{20}H_{34}O_6$	$C_{31}H_{58}N_2O_5$	539.4418
	2,3-dinor-6-keto-PGF1α	$C_{18}H_{30}O_6$	$C_{29}H_{54}N_2O_5$	511.4105
	11-dehydro-TXB2	$C_{20}H_{32}O_6$	$C_{31}H_{56}N_2O_5$	537.4262
	12(S)-HHTrE	$C_{17}H_{28}O_3$	$C_{28}H_{52}N_2O_2$	449.4102
Amino acids	Alanine (Ala)	$C_3H_7NO_2$	$C_{14}H_{31}N_3O$	258.2540
	Arginine (Arg)	$C_6H_{14}N_4O_2$	$C_{17}H_{38}N_6O$	343.3180
	Aspartic acid (Asp)	$C_4H_7NO_4$	$C_{15}H_{31}N_3O_3$	302.2438
	Cysteine (Cys)	$C_3H_7NO_2S$	$C_{14}H_{31}N_3OS$	290.2261
	Glutamic acid (Glu)	$C_5H_9NO_4$	$C_{16}H_{33}N_3O_3$	316.2595
	Glycine (Gly)	$C_2H_5NO_2$	$C_{13}H_{29}N_3O$	244.2383
	Histidine (His)	$C_6H_9N_3O_2$	$C_{17}H_{33}N_5O$	324.2758
	Isoleucine (Ile)	$C_6H_{13}NO_2$	$C_{17}H_{37}N_3O$	300.3009
	Leucine (Leu)	$C_6H_{13}NO_2$	$C_{17}H_{37}N_3O$	300.3009
	Lysine (Lys)	$C_6H_{14}N_2O_2$	$C_{17}H_{38}N_4O$	315.3129
	Methionine (Met)	$C_5H_{11}NO_2S$	$C_{16}H_{35}N_3OS$	318.2574
	Phenylalanine (Phe)	$C_9H_{11}NO_2$	$C_{20}H_{35}N_3O$	334.2853
	Proline (Pro)	$C_5H_9NO_2$	$C_{16}H_{33}N_3O$	284.2696
	Serine (Ser)	$C_3H_7NO_3$	$C_{14}H_{31}N_3O_2$	274.2489
	Threonine (Thr)	$C_4H_9NO_3$	$C_{15}H_{33}N_3O_2$	288.2646
	Tryptophan (Trp)	$C_{11}H_{12}N_2O_2$	$C_{22}H_{36}N_4O$	373.2962
	Tyrosine (Tyr)	$C_9H_{11}NO_3$	$C_{20}H_{35}N_3O_2$	350.2802

	Valine (Val)	$C_5H_{11}NO_2$	$C_{16}H_{35}N_3O$	286.2853
	Asparagine (Asn)	$C_4H_8N_2O_3$	$C_{15}H_{32}N_4O_2$	301.2598
	Glutamine (Gln)	$C_5H_{10}N_2O_3$	$C_{16}H_{34}N_4O_2$	315.2755
TCA intermediates	Aconitic acid	$C_6H_6O_6$	$C_{17}H_{30}N_2O_5$	343.2227
	Oxaloacetic acid (OAA)	$C_4H_4O_5$	$C_{15}H_{28}N_2O_4$	301.2122
	Isocitric acid (Isocit)	$C_6H_8O_7$	$C_{17}H_{32}N_2O_6$	361.2333
	Citric acid (Cit)	$C_6H_8O_7$	$C_{17}H_{32}N_2O_6$	361.2333
	Malic acid (Mal)	$C_4H_6O_5$	$C_{15}H_{30}N_2O_4$	303.2278
	Fumaric acid (Fum)	$C_4H_4O_4$	$C_{15}H_{28}N_2O_3$	285.2173
	alpha-Ketoglutaric acid (α-KG)	$C_5H_6O_5$	$C_{16}H_{30}N_2O_4$	315.2278
	Succinic acid (Suc)	$C_4H_6O_4$	$C_{15}H_{30}N_2O_3$	287.2329
Pyruvate metabolites	Lactic acid (Lac)	$C_3H_6O_3$	$C_{14}H_{30}N_2O_2$	259.2380
SCFAs	Propionic acid	$C_3H_6O_2$	$C_{14}H_{30}N_2O$	243.2431
	Isobutyric acid	$C_4H_8O_2$	$C_{15}H_{32}N_2O$	257.2587
	Butyric acid	$C_4H_8O_2$	$C_{15}H_{32}N_2O$	257.2587
	Isovaleric acid	$C_5H_{10}O_2$	$C_{16}H_{34}N_2O$	271.2744
	Valeric acid	$C_5H_{10}O_2$	$C_{16}H_{34}N_2O$	271.2744

TABLE S2 | Identified differential metabolites in the serum of AADs patients by untargeted metabolomics

Primary	Identification	HMDB ID	VIP value	Rt (min)	Selected ion	Experimental *m/z*	Derivatization formula	Chemical formula	MS/MS
04809	(2S)-2,5-diamino-5-oxopentanoic acid; phosphane		3.90	1.1	$[M+H]^+$	349.2702	C16H37N4O2P	C5H13N2O3P	86.0960, 128.1046, 158.1175, 212.1280, 229.2270, 248.1484, 313.2485
03850	Unidentified		1.62	6.4	$[M+H]^+$	331.2609	—	C17H34N2O4	98.0962, 212.1285, 230.1389, 313.2479
01133	Lactic acid*	HMDB0000190	3.28	7.4	$[M+H]^+$	259.2395	C14H30N2O2	C3H6O3	86.0960, 158.1177, 175.1499, 217.1912
06333	Unidentified		1.52	7.7	$[M+H]^+$	381.3965	—	C23H48N4	154.1595, 170.1932, 253.2638, 354.3846
01487	3-Hydroxybutyric acid*	HMDB0000011	2.05	8.0	$[M+H]^+$	273.2548	C15H32N2O2	C4H8O3	86.0965, 1228.1432, 172.1319, 187.1808, 231.2065
06015	Hydroxynonanedioic acid*	HMDB0000784	2.48	15.4	$[M+H]^+$	373.3073	C20H40N2O4	C9H16O5	86.0968, 128.1444, 151.0770, 236.1670, 313.2515, 331.2611
05039	4-O-Methylgallic acid	HMDB0013198	1.56	16.6	$[M+H]^+$	353.2452	C19H32N2O4	C8H8O5	126.1279, 128.1437, 152.1072, 211.1817, 253.2280, 252.1239, 311.1974
06014	Hydroxynonanedioic acid isomer*		2.09	16.8	$[M+H]^+$	373.3081	C20H40N2O4	C9H16O5	86.0968, 128.1439, 168.1379, 196.1338, 331.2603
01432	Isovaleric acid*	HMDB0000718	2.80	17.0	$[M+H]^+$	271.2764	C16H34N2O	C5H10O2	86.0966, 128.1438, 145.1705, 170.1545,

									187.1821, 229.2279
12925	N-Myristoyl Lysine	HMDB0242055	2.12	18.4	$[M+H]^{+}$, $[M+2H]^{++}$	525.5114, 263.2604	C31H64N4O2	C20H40N2O3	128.1441, 382.3438, 399.3711, 441.4168, 483.4631, 525.5087
02132	Benzoic acid*	HMDB0001870	3.10	18.6	$[M+H]^{+}$	291.2445	C18H30N2O	C7H6O2	105.0334, 128.1439, 190.1235, 207.1499, 249.1971, 291.2439
04108	Melanic acid*	HMDB00130	2.36	18.9	$[M+H]^{+}$	337.2503	C19H32N2O3	C8H8O4	126.1268, 128.1425, 152.1062, 211.1807, 236.1277, 295.2009
06621	Hydroxydecanedioic acid*	HMDB0000350	2.38	19.0	$[M+H]^{+}$	387.3231	C21H42N2O4	C10H18O5	86.0968, 128.1443, 182.1194, 241.1927, 259.2038, 345.2772
05255	Nonanedioic acid	HMDB0000784	2.90	19.8	$[M+H]^{+}$	357.3128	C20H40N2O3	C9H16O4	69,0707, 86.0960, 128.1443, 152.1079, 238.1818, 315.2651
04593	Indole-3-acetic acid*	HMDB0000197	1.63	20.0	$[M+H]^{+}$	344.2714	C21H33N3O	C10H9NO2	86.0960, 130.0647, 158.0601, 243.1492, 260.1752, 302.2234
07245	Hydroxyundecanedioic acid*	HMDB0000888	2.78	20.1	$[M+H]^{+}$	401.3388	C22H44N2O4	C11H20O5	128.1428, 196.1329, 273.2169, 359.2903
04574	Hydroxynonanoic acid*	HMDB0031513	1.75	21.0	$[M+H]^{+}$	343.3333	C20H42N2O2	C9H18O3	86.0964, 128.1434, 224.2005, 283.2747, 301.2852
05308	3-Indolepropionic acid*	HMDB0002302	2.07	21.5	$[M+H]^{+}$	358.2863	C22H35N3O	C11H11NO2	86.0957, 130.0646, 172.0758, 257.1649,

									326.2386
07865	Hydroxydodecanedioic acid*	HMDB0000413	5.26	21.5	$[M+H]^{+}$	415.3548	C23H46N2O4	C12H22O5	128.1435, 238.1806, 315.2646, 373.3056
08577	3-hydroxy-2-octyl-glutaric acid		2.19	23.4	$[M+H]^{+}$	429.3685	C24H48N2O4	C13H24O5	86.0972, 128.1443, 224.1657, 301.2496, 387.3228
00598	Dibutyl malate	HMDB0031696	1.94	23.7	$[M+H-H2O]^{+}$	229.1453	—	C12H22O5	69.0699, 125.0593, 143.0724, 229.1452
02996	Octanoic acid*	HMDB0000482	1.51	24.4	$[M+H]^{+}$	313.3206	C19H40N2O	C8H16O2	57.0697, 86.0959, 212.2012, 271.2750
09228	Hydroxytetradecanedioic acid*	HMDB0000394	5.43	25.6	$[M+H]^{+}$	443.3856	C25H50N2O4	C14H26O5	128.1433, 238.1814, 315.2654, 401.3371
10020	Hydroxypentadecanedioic acid*	HMDB0031885	1.72	28.1	$[M+H]^{+}$	457.4003	C26H52N2O4	C15H28O5	86.0968, 128.1433, 166.1221, 252.1966, 329.2779, 415.3532
01058	Glycerolipids		2.46	29.6	$[M+H]^{+}$, $[M+H-H2O]^{+}$	275.1852, 257.1735	—	C14H26O5	97.1013, 125.0962, 171.1014, 239.1643, 257.1749
03545	Pelargonic acid*	HMDB0000847	2.45	30.1	$[M+H]^{+}$	327.3378	C20H42N2O	C9H18O2	86.0958, 128.1427, 141.1267, 226.2161, 243.2428, 285.2892
10600	Hydroxy-hexadecandioic acid*		2.01	30.1	$[M+H]^{+}$	471.4173	C27H54N2O4	C16H30O5	128.1430, 180.1375, 266.2111, 429.3676
06803	Diglyceride		1.57	30.5	$[M+2H]^{++}$,	391.3436,	C50H88N2O4	C39H64O5	86.0968, 128.1433, 152.1070, 443.3850,

					$[M+H]^+$	781.6787			552.4364, 697.5838, 739.6281
08740	Unidentified		2.41	31.2	$[M+H]^+$	432.3129	—	C26H41NO4	147.1171, 175.1461, 215.1778, 321.2578, 339.2677, 414.3001
16841	Unidentified		1.92	36.5	$[M+H]^+$	671.5216	C37H70N2O8	C26H46O9	128.1446, 238.1798, 315.2180, 355.2940, 397.3459, 629.4750, 671.5210
06880	Aminophenyl-dioxobutanoic acid*	HMDB0000978	2.59	36.6	$[M+NH4]^+$, $[M+H]^+$	393.2873, 376.2609	C21H33N3O3	C10H9NO4	125.0718, 209.1306, 218.1293, 275.1769, 292.2034, 302.1878
17308	Unidentified		2.62	38.5	$[M+H]^+$	699.5532	C39H74N2O8	C28H50O9	128.1434, 152.1071, 238.1812, 401.3390, 657.5052, 699.5502
11307	5(S)-HETE*	HMDB0011134	1.63	38.9	$[M+H]^+$	489.4426	C31H56N2O2	C20H32O3	86.0972, 429.3854, 471.4311
12556	LysoPC(18:3(9Z,12Z,15Z)/0:0)	HMDB0010388	1.83	39.4	$[M+H]^+$	518.3264	—	C26H48NO7P	86.0971, 104.1086, 124.9998, 184.0737, 500.3138
11485	LysoPC(16:1(9Z)/0:0)	HMDB0010383	1.84	39.7	$[M+H]^+$	494.325	—	C24H48NO7P	86.0971, 104.1075, 125.0011, 184.0741, 476.3135
07071	Myristic acid*	HMDB0000806	1.80	40.0	$[M+H]^+$	397.4159	C25H52N2O	C14H28O2	86.0966, 128.1435, 296.2952, 355.3680
09438	Linolenic acid*	HMDB0001388	1.88	40.2	$[M+H]^+$	447.4333	C29H54N2O	C18H30O2	86.0964, 128.1434, 405.3830
12658	LysoPC(18:2(9Z,12Z)/0:0)	HMDB0010386	6.72	40.4	$[M+H]^+$	520.3402	—	C26H50NO7P	86.0971, 104.1067, 124.9995, 184.0728,

									229.1434, 258.1106, 337.2734, 502.3266
13659	LysoPC(20:4(5Z,8Z,11Z,14Z)/0:0)	HMDB0010395	4.19	40.5	$[M+H]^+$	544.341	—	C28H50NO7P	86.0971, 104.1071, 125.0000, 184.0726, 258.1103, 526.3283
14426	LysoPC(22:6(4Z,7Z,10Z,13Z,16Z,19Z)/0:0)	HMDB0010404	2.53	40.5	$[M+H]^+$	568.3418	—	C30H50NO7P	86.0971, 104.1071, 125.0001, 184.0739, 550.3304
09520	9,11-octadecadienoic acid*	HMDB0005047	3.47	40.7	$[M+H]^+$	449.4468	C29H56N2O	C18H32O2	86.0970, 348.3263, 407.3985
10684	Arachidonic acid*	HMDB0001043	2.73	40.7	$[M+H]^+$	473.4477	C31H56N2O	C20H32O2	86.0968, 128.1433, 431.3978
11656	Docosahexaenoic acid*	HMDB0002183	1.65	40.7	$[M+H]^+$	497.4478	C33H56N2O	C22H32O2	86.0965, 125.0001, 128.1435, 184.0736, 455.4007
14492	LysoPC(22:5(4Z,7Z,10Z,13Z,16Z)/0:0)	HMDB0010402	1.81	40.8	$[M+H]^+$	570.3573	—	C30H52NO7P	86.0969, 104.1071, 125.0002, 184.0736, 552.3431
18840	PC(16:0/22:4(7Z,10Z,13Z,16Z)) or isomer	HMDB0007988	1.82	40.8	$[M+H]^+$	810.6029	—	C46H84NO8P	125.0001, 184.0732, 810.5979
08322	Palmitic acid*	HMDB0000220	3.36	40.9	$[M+H]^+$	425.4466	C27H56N2O	C16H32O2	86.0963, 128.1433, 145.1709, 239.2371, 324.3262, 341.3533, 383.3985
11588	LysoPC(16:0/0:0)	HMDB0010382	6.45	40.9	$[M+H]^+$	496.339	—	C24H50NO7P	86.0971, 125.0001, 184.0736, 478.3291
09643	Oleic acid*	HMDB0000207	4.02	41.0	$[M+H]^+$	451.4621	C29H58N2O	C18H34O2	86.0957, 128.1433, 350.3412, 367.3688,

									409.4145
13726	LysoPC(20:3(8Z,11Z,14Z)/0:0)	HMDB0010394	2.26	41.0	$[M+H]^+$	546.3568	—	C28H52NO7P	86.0971, 104.1071, 125.0001, 184.0736, 258.1103, 528.3457
12774	LysoPC(18:1(9Z)/0:0)	HMDB0002815	5.39	41.3	$[M+H]^+$	522.3558	—	C26H52NO7P	86.0971, 104.1067, 125.0000, 184.0734, 504.3452
09775	Stearic acid*	HMDB0000827	3.34	41.4	$[M+H]^+$	453.4782	C29H60N2O	C18H36O2	86.0956, 128.1433, 352.3565, 411.4295
12224	LysoPC(17:0/0:0)	HMDB0012108	1.86	41.5	$[M+H]^+$	510.3557	—	C25H52NO7P	86.0971, 104.1086, 124.9998, 184.0741, 492.3467
06781	Bis(2-ethylhexyl) phthalate	HMDB0249243	2.02	41.8	$[M+H]^+$	391.2856	—	C24H38O4	121.0265, 149.0225, 167.0325, 279.1572
17331	SM(d18:1/16:1(9Z))	HMDB0240613	2.44	41.8	$[M+H]^+$	701.5613	—	C39H77N2O6P	86.0975, 124.9997, 184.0736, 683.5514
18148	PC(14:0/20:2(11Z,14Z)) or isomer	HMDB0007880	25.20	42.0	$[M+H]^+$	758.5712	—	C42H80NO8P	124.9997, 184.0735, 758.5694
18792	PC(16:0/22:6(4Z,7Z,10Z,13Z,16Z,19Z)) or isomer	HMDB0007991	11.14	42.0	$[M+H]^+$	806.5709	—	C46H80NO8P	124.9995, 184.0733，806.5667
12869	LysoPC(18:0/0:0)	HMDB0010384	8.61	42.1	$[M+H]^+$	524.3712	—	C26H54NO7P	86.0971, 104.1062, 125.0000, 184.0726, 258.1103, 341.3046, 506.3585
08809	Unidentified		2.11	42.3	$[M+2H]^{++}$, $[M+H]^+$	433.3926, 865.7762	—	C47H100N4O9	269.2952, 369.3524, 709.6612, 764.6544

11128	LysoPG(16:0/0:0)	HMDB0240601	1.80	42.3	$[M+H]^+$	485.2923	—	C22H45O9P	91.0639, 133.1099, 147.0804
04081	Fatty amides		1.81	42.4	$[M+H]^+$	336.3273	—	C22H41NO	81.0700, 95.0864, 109.1019, 121.1016, 147.1167, 161.1339, 301.2908, 319.2992
11654	Unidentified		1.67	42.4	$[M+NH4]^+$	497.4325	—	C29H53NO4	155.1065, 187.2172, 210.1856, 211.1332, 282.2077, 293.2104, 311.2226, 396.3120
12299	LysoPC(18:0/0:0) isomer	HMDB0010384	1.77	42.4	$[M+H]^+$	524.3725	—	C26H54NO7P	104.1086, 124.9998, 184.0727, 258.1106, 341.3052, 506.3591
13858	LysoPC(20:1(11Z)/0:0)	HMDB0010391	1.77	42.4	$[M+H]^+$	550.3873	—	C28H56NO7P	104.1071, 125.0001, 184.0730, 528.3771, 532.3764
04172	13-Docosenamide	HMDB0244507	6.90	42.6	$[M+H]^+$, $[2M+H]^+$	338.3426, 675.6771	—	C22H43NO	83.0859, 97.1013, 135.1174, 149.1331, 163.1495, 303.3053, 321.3158
09423	Didecyl phthalate or isomer	HMDB0251217	1.77	42.6	$[M+H]^+$	447.3488	—	C28H46O4	149.0241
16463	1-Palmitoyl-2-(9-oxo-nonanoyl)-sn-glycero-3-phosphocholine		1.70	42.7	$[M+H]^+$	650.439	—	C33H64NO9P	124.9996, 184.0732, 650.4374
13209	Unidentified		2.36	42.9	$[M+H]^+$	532.3565	—	C31H50NO4P	263.0463, 319.1125, 375.1725, 431.2324, 487.2976
18293	SM(d16:1/20:5(7Z,9Z,11E,13E,17Z)	HMDB0290285	2.92	42.9	$[M+H]^+$	769.5112	—	C41H73N2O9P	184.0736, 239.1649, 257.1762, 427.2682,

	-3OH(5,6,15))								513.3418, 683.4346
01852	(2S,3R)-2-Amino-4-octadecene-3-ol	HMDB0242695	1.54	43.1	$[M+H]^+$	284.2958	—	C18H37NO	84.0788, 266.2862
17366	SM(d18:1/16:0)	HMDB0010169	1.83	43.1	$[M+H]^+$	703.5762	—	C39H79N2O6P	124.9997, 184.0740, 338.3422
03862	MG(16:0/0:0/0:0)	HMDB0011564	1.51	43.2	$[M+H]^+$	331.2854	—	C19H38O4	71.0858, 95.0857, 109.1016, 123.1182, 239.2391, 257.2491
05760	Vitamin d derivative		4.29	43.2	$[M+H]^+$	369.3532	—	C27H44	95.0865, 133.1025, 135.1178, 147.1181, 161.1336, 175.1497, 215.1811, 243.2117, 257.2306
13230	Unidentified		1.92	43.4	$[M+H]^+$	533.1941	—	C27H33O9P	161.0962, 175.1121, 231.1747, 382.1827

73 metabolites were identified with no false positive; metabolites with*: mean that they were confirmed by comparing the exact mass data, MS fragmentation, and retention time with standards; VIP value: variable importance for the projection.

TABLE S3 | Total differential metabolites between AAD patients and healthy controls by targeted and untargeted metabolomics

Class	Name	HMDB_ID	Normal VS AR			Normal VS Asthma			Normal VS CARAS			Total_Kruskal-Wallis Test	
			P	log2FC	VIP	P	log2FC	VIP	*P*	log2FC	VIP	P	VIP
Peptide	1-(Hydroxymethyl)-5-oxo-L-proline		0.045	-0.189	0.919	0.147	-0.287	1.303	0.290	-0.069	1.367	0.092	1.449
Fatty acid	12 (S)-HHTrE	HMDB0012535	0.126	-0.463	0.597	0.170	-0.420	0.247	0.238	-0.218	0.152	0.307	0.268
Fatty acid	12(S)-HEPE	HMDB0010202	0.492	0.274	0.772	0.385	0.252	0.038	0.063	0.542	0.061	0.094	0.053
Fatty acid	12a-hydroxy-3-oxocholadienic acid	HMDB0000385	0.037	0.879	0.858	0.643	0.627	0.067	0.032	0.667	0.065	0.068	0.084
Fatty acid	12-hydroxy-8,10-octadecadienoic acid or isomer	HMDB0029998	0.526	0.631	0.819	0.685	0.927	0.420	0.273	0.616	0.325	0.783	0.247
Fatty acid	13(S)-HOTrE	HMDB0010203	0.025	1.506	0.892	0.023	1.457	0.164	0.006	1.221	0.120	0.058	0.099
Fatty acid	13(S)-HPODE	HMDB0003871	0.342	0.032	0.748	0.440	-0.195	0.076	0.424	0.240	0.070	0.007	0.114
Fatty acid	13,14-dihydro Prostaglandin F1α	HMDB0244516	0.003	0.962	1.302	0.000	1.064	0.170	0.000	1.376	0.176	0.000	0.129
Fatty acid	13-HODE or isomer	HMDB0004667	0.864	0.716	0.773	0.671	0.849	0.365	0.768	0.544	0.295	0.887	0.226
	13-HODE or isomer-2		0.273	0.279	0.626	0.049	0.337	0.142	0.006	0.486	0.143	0.016	0.144
Fatty acid	15(S)-HEPE	HMDB0010209	0.000	0.935	1.218	0.000	1.012	0.059	0.000	1.320	0.075	0.000	0.054
Fatty acid	15(S)-HETE	HMDB0003876	0.721	0.674	0.708	0.202	0.908	0.213	0.313	0.657	0.184	0.433	0.124

Fatty acid	15(S)-HETrE	HMDB0005045	0.853	-0.024	0.440	0.602	-0.013	0.135	0.834	-0.051	0.123	0.729	0.144
Fatty acid	15-methyl palmitic acid	HMDB0061709	0.035	0.366	0.769	0.066	0.336	0.905	0.006	0.562	1.179	0.016	1.020
Peptide	2-(Carboxymethyl-(2-(4-hydroxyphenyl)ethyl)amino)acetic acid		0.004	1.379	1.001	0.082	0.565	0.349	0.004	1.052	0.412	0.003	0.781
Fatty acid	2,3-dinor-6-keto-PGF1a	HMDB0062618	0.005	1.537	1.072	0.000	2.490	0.066	0.000	3.288	0.088	0.000	0.073
Carboxylic acid	2,6-Diaminopimelic acid	HMDB0001370	0.535	0.298	0.657	0.306	0.187	0.186	0.641	0.311	0.198	0.681	0.237
Organic acid	2-[2-ethoxy-4-(hydroxymethyl)phenoxy]pentanoic acid		0.070	0.406	0.901	0.549	0.149	0.223	0.009	0.667	0.506	0.005	0.618
	2-dodecenoic acid or isomer	HMDB0340653	0.937	-0.414	0.629	0.196	-0.993	0.206	0.977	-0.326	0.143	0.071	0.233
Fatty acid	2-Hydroxyoctanoic acid	HMDB0000711	0.015	0.655	1.219	0.072	0.478	0.719	0.007	0.684	0.773	0.031	0.648
Fatty acid	2-lauroleic acid or isomer	HMDB0010729	0.484	0.121	0.467	0.714	0.276	0.207	0.717	0.146	0.149	0.852	0.154
Keto acid	2-Oxoglutaramate	HMDB0001552	0.644	0.044	0.410	0.122	-0.297	0.051	0.308	-0.134	0.041	0.134	0.091
Keto acid	2-Oxo-isocaproate	HMDB0000695	0.251	0.490	0.530	0.728	-0.168	0.130	0.238	0.366	0.152	0.012	0.413
	2-Oxo-isocaproate isomer		0.362	-0.100	0.509	0.000	-0.819	0.488	0.065	-0.276	0.134	0.000	0.509
Fatty acid ester	3-[(4-Carboxybutanoyl)oxy]-4-(trimethylazaniumyl)butanoate	HMDB0240779	0.509	0.136	0.510	0.817	-0.098	0.250	0.341	0.280	0.393	0.292	0.441
Fatty acid	3-Acetyl-6-oxo-heptanoic acid	HMDB0340970	0.000	1.305	1.478	0.002	0.881	1.001	0.000	1.190	1.033	0.000	0.914
	3-Acetyl-6-oxo-heptanoic acid isomer		0.001	1.105	1.202	0.031	0.539	0.453	0.005	0.804	0.516	0.002	0.601

Peptide	3-Amino-3-(3-cyclohexenyl)propanoic acid		0.005	0.784	1.265	0.020	0.568	0.687	0.001	0.982	0.873	0.002	0.665
	3-dodecenoic acid or isomer	HMDB0340654	0.037	0.615	0.753	0.239	0.269	0.350	0.130	0.459	0.408	0.157	0.298
Beta hydroxy acid	3-Hydroxybutyric acid	HMDB0000011	0.692	-0.118	0.425	0.035	-0.693	2.570	0.024	-0.568	2.095	0.001	2.021
Beta hydroxy acid	3-Hydroxybutyric acid isomer		0.853	0.051	0.679	0.056	-0.449	1.157	0.156	-0.263	0.726	0.013	1.249
Hydroxy acid	3-Hydroxyoctanoic acid	HMDB0001954	0.979	-0.262	0.493	0.395	-0.386	0.217	0.819	-0.183	0.120	0.302	0.193
Indolyl carboxylic acid	3-Indolepropionic acid	HMDB0002302	0.061	-1.779	0.609	0.029	-1.503	2.011	0.051	-2.498	1.948	0.096	1.859
Keto acid	3-Oxodecanoic acid	HMDB0010724	0.006	0.695	1.307	0.043	0.583	0.269	0.002	0.794	0.307	0.008	0.249
Fatty acid	4,7,10,13-docosatetraenoic acid	HMDB0002226	0.006	-0.475	1.256	0.004	-0.517	0.998	0.351	-0.081	0.539	0.000	0.972
Fatty acid	5(S)-HEPE	HMDB0005081	0.000	1.122	1.325	0.001	1.002	0.113	0.000	1.447	0.157	0.000	0.118
Fatty acid	5(S)-HETE	HMDB0011134	0.219	2.236	0.835	0.018	2.524	0.997	0.004	2.066	0.826	0.014	0.558
Fatty acid	5,9,12-octadecatrienoic acid or isomer	HMDB0256562	0.916	0.085	0.475	0.602	-0.098	1.344	0.435	0.361	1.832	0.192	2.200
Amino acid	5-Oxopline	HMDB0246561	0.003	-0.874	1.804	0.002	-0.951	1.342	0.005	-0.765	0.934	0.010	0.937
Fatty acid	7,10-Hexadecadienoic acid	HMDB0000477	0.802	-0.003	0.674	0.463	-0.098	0.314	0.574	0.215	0.360	0.374	0.447
Fatty acid	8(S)-HETE	HMDB0004679	0.968	-0.236	0.367	0.671	-0.256	0.554	0.939	-0.214	0.509	0.764	0.654
Fatty acid	9(S)-HPODE	HMDB0006940	0.501	0.589	0.673	0.562	0.161	0.070	0.099	0.442	0.079	0.187	0.086

Fatty Acyl	9,11-octadecadienoic acid	HMDB0005047	0.958	-0.036	0.731	0.499	0.046	3.485	0.098	0.272	4.433	0.006	4.754
Keto acid	Acetamido-oxohexanoate	HMDB0012150	0.003	1.594	0.830	0.079	0.659	0.591	0.007	1.026	0.643	0.002	1.319
Carboxylic acid	Acetic acid	HMDB0000042	0.006	-0.443	1.250	0.000	-0.689	3.327	0.001	-0.525	2.155	0.001	2.376
Short-chain keto acid	Acetoacetate	HMDB0000060	0.535	0.099	0.496	0.113	-0.243	0.137	0.689	-0.020	0.102	0.045	0.159
Short-chain keto acid	Acetolactate	HMDB0006833	0.006	-2.506	1.854	0.002	-2.368	0.537	0.009	-2.380	0.466	0.002	0.463
Fatty acid ester	Acetylcarnitine	HMDB0000201	0.003	1.049	0.817	0.034	0.224	0.357	0.002	0.873	0.503	0.002	0.916
Fatty acid ester	Acetylcarnitine isomer		0.009	1.087	0.871	0.122	0.359	0.275	0.010	0.883	0.354	0.010	0.678
Amino acid	Acetyllysine	HMDB0000446	0.010	0.378	0.800	0.109	0.202	0.635	0.162	0.305	0.707	0.044	0.829
Amino acid	Acetylproline	HMDB0094701	0.009	-1.344	1.238	0.000	-1.818	0.906	0.000	-1.680	0.757	0.000	0.646
Amino acid	Acetylserine	HMDB0003011	0.874	0.089	0.865	0.406	-0.184	0.101	0.962	0.000	0.081	0.405	0.072
TCA metabolites	Aconitic acid	HMDB0000072	0.428	-0.335	0.421	0.602	-0.323	0.014	0.864	-0.106	0.008	0.328	0.016
Fatty acid	Adipic acid	HMDB0000448	0.036	0.683	1.164	0.039	0.621	0.309	0.005	0.817	0.343	0.018	0.272
Fatty acid	Adipic acid isomer		0.937	0.161	0.922	0.938	0.294	0.260	0.954	0.084	0.210	0.974	0.220
Gamma-keto acid	a-ketoglutaric acid	HMDB0000208	0.692	-0.330	0.459	1.000	-0.600	0.134	0.827	-0.640	0.121	0.262	0.093
Peptide	Ala Ile Lys Asn		0.262	0.698	0.960	0.003	1.167	1.224	0.001	1.391	1.160	0.000	0.920
Peptide	Ala Lys Gln Thr		0.000	1.244	1.475	0.000	1.390	0.349	0.000	1.793	0.367	0.000	0.277

Amino acid	Alanine	HMDB0000161	0.303	0.279	0.526	0.334	0.219	0.323	0.162	0.289	0.359	0.599	0.545
Amino acid	Alanine or isomer		0.088	-0.858	1.637	0.003	-1.306	4.137	0.004	-1.183	3.343	0.000	3.198
Amino acid	Alanylleucine	HMDB0028691	0.015	0.508	0.897	0.069	0.350	0.590	0.029	0.524	0.625	0.092	0.617
Amino acid	Alanylleucine isomer	HMDB0028691	0.039	0.379	0.803	0.147	0.221	0.505	0.101	0.395	0.587	0.218	0.601
Amino acid	Allylglycine	HMDB0251510	0.018	0.756	0.813	0.279	0.263	0.393	0.205	0.373	0.353	0.004	0.604
Amino acid	Amino-acetoacetate	HMDB0006454	0.045	0.365	0.595	0.562	-0.029	0.183	0.282	0.056	0.171	0.003	0.253
Amino acid	Aminoacrylic acid	HMDB0003609	0.518	-0.006	0.534	0.575	-0.069	0.121	0.827	0.031	0.149	0.817	0.189
Amino acid	Aminoadipate	HMDB0000510	0.119	-0.286	1.056	0.463	-0.309	0.797	0.322	-0.153	0.766	0.060	0.743
Amino acid	Aminobutyric acid	HMDB0000112	0.002	-0.719	1.643	0.037	-0.554	0.190	0.005	-0.621	0.163	0.001	0.143
Amino acid	Aminoheptanedioic acid	HMDB0034252	0.066	-0.120	1.038	0.395	-0.213	0.812	0.290	0.040	0.868	0.045	0.800
Fatty acid	Aminoheptanoic acid	HMDB0247233	0.009	0.605	1.029	0.025	0.488	0.882	0.002	0.716	1.068	0.006	0.835
Fatty acid	Aminoheptanoic acid isomer	HMDB0242128	0.022	0.360	0.704	0.005	0.429	0.935	0.001	0.583	1.049	0.001	0.868
Hydroxy acid	Amino-hydroxydodecanoic acid		0.182	-0.134	0.923	0.001	-0.416	0.585	0.112	-0.167	0.349	0.005	0.462
Fatty acid	Aminoicosenoic acid		0.588	0.070	0.531	0.657	0.126	0.128	0.192	0.387	0.151	0.339	0.149
Amino acid	Aminomalonic acid	HMDB0001147	0.229	-0.374	1.032	0.728	-0.534	0.563	0.245	-0.585	0.513	0.086	0.499
Organic acid	Aminophenyl-dioxobutanoic acid	HMDB0000978	0.000	-0.875	1.693	0.137	-0.325	1.397	0.725	-0.062	0.999	0.000	2.245

Benzoic acid	Anthranilic acid	HMDB0001123	0.240	0.204	0.872	0.076	0.375	0.153	0.002	0.831	0.211	0.000	0.216
Fatty acid	Arachidonic acid	HMDB0001043	0.000	-0.665	1.684	0.002	-0.567	3.805	0.112	-0.163	1.781	0.000	3.117
Fatty acid	Arachidoyl glycine		0.004	-0.710	1.018	0.024	-0.496	0.129	0.049	-0.393	0.092	0.029	0.118
	Arachidoyl glycine isomer		0.002	-0.707	1.107	0.011	-0.499	0.147	0.046	-0.336	0.098	0.007	0.131
Peptide	Arg Ala Ala		0.000	1.560	1.603	0.000	1.310	0.708	0.000	1.744	0.820	0.000	0.612
Amino acid	Arginine	HMDB0000517	0.025	0.566	0.780	0.246	0.330	0.118	0.059	0.416	0.104	0.056	0.151
Peptide	Asp Ile Lys Lys		0.000	2.302	1.653	0.000	1.936	1.173	0.000	2.652	1.464	0.000	1.057
Amino acid	Asparagine	HMDB0000168	0.229	0.188	0.579	0.216	-0.251	0.151	0.561	-0.102	0.088	0.000	0.235
Amino acid	Aspartic acid	HMDB0000191	0.136	-0.043	1.002	0.714	-0.247	0.712	0.346	-0.219	0.630	0.192	0.671
Fatty acid	Behenic acid	HMDB0000944	0.552	-0.033	0.304	0.246	0.184	0.129	0.034	0.651	0.389	0.002	0.384
Benzoic acid	Benzeneacetate	HMDB0000209	0.251	0.331	0.731	0.001	1.129	1.325	0.000	1.467	1.530	0.000	1.392
Benzoic acid	Benzoic acid	HMDB0001870	0.009	0.683	0.933	0.364	-2.053	1.786	0.391	-2.033	1.439	0.000	2.700
Fatty acid	Butyric acid	HMDB0000039	0.885	-0.367	0.395	0.142	-1.017	0.672	0.654	-0.374	0.534	0.036	0.836
Peptide	Caproyl glutamic acid		0.016	1.467	1.098	0.032	1.207	0.303	0.002	1.481	0.290	0.011	0.274
Peptide	Caproyl glutamic acid isomer		0.012	1.506	1.080	0.037	1.183	0.289	0.002	1.455	0.265	0.011	0.250

Amino acid	Carbamyl-aspartate	HMDB0000828	0.027	-0.097	0.973	0.487	0.058	0.167	0.227	-0.003	0.143	0.019	0.150
Bile acid	Chenodeoxycholic acid	HMDB0000518	0.328	0.426	0.359	0.354	0.446	0.283	0.101	0.710	0.298	0.327	0.268
Bile acid	Cholestenoic acid	HMDB0012453	0.682	0.145	0.851	0.923	0.011	0.160	0.000	0.671	0.464	0.000	0.470
Bile acid	Cholic acid	HMDB0000619	0.018	0.853	0.828	0.938	-0.043	0.074	0.046	0.813	0.217	0.000	0.352
Fatty acid	Citraconic acid	HMDB0000634	0.812	-0.013	0.453	0.315	-0.235	0.146	0.135	0.699	0.205	0.005	0.281
Amino acid	Citrulline	HMDB0000904	0.054	-0.133	1.037	0.406	-0.205	0.657	0.269	0.018	0.691	0.030	0.641
Amino acid	Creatine	HMDB0000064	0.001	-1.414	1.762	0.000	-1.935	0.254	0.000	-1.362	0.175	0.000	0.187
Organic acid	Cyano-aminobutyric acid		0.022	0.540	0.443	0.549	-0.262	0.093	0.164	-0.005	0.043	0.001	0.222
Fatty acid	cyanononanoic acid		0.003	0.873	1.259	0.018	0.662	0.174	0.001	1.135	0.225	0.001	0.168
Amino acid	Cysteic acid	HMDB0002757	0.452	0.335	0.874	0.877	0.093	0.460	0.725	0.025	0.377	0.289	0.603
Amino acid	Cysteine sulfinate	HMDB0000996	0.001	1.329	0.967	0.013	1.508	0.112	0.008	1.270	0.098	0.010	0.073
Fatty acid	Decadienoic acid		0.761	0.986	0.279	0.132	-0.018	0.101	0.205	0.348	0.215	0.001	0.440
Fatty acid	Decanoic acid	HMDB0000511	0.086	0.694	0.491	0.216	0.751	1.349	0.030	0.406	0.857	0.196	1.427
Peptide	Decanoyl-aspartic acid		0.013	1.357	1.120	0.037	1.077	0.436	0.003	1.342	0.397	0.017	0.397
Fatty acid	Decatrienoic acid	HMDB0035235	0.309	0.007	0.268	0.061	-0.288	0.345	0.475	0.119	0.192	0.266	0.517
	Decatrienoic acid isomer		0.267	0.637	0.368	0.892	0.138	0.209	0.063	0.611	0.373	0.030	0.513

Fatty acid	Decenedioic acid	HMDB0000603	0.070	0.639	1.103	0.032	0.594	0.487	0.005	0.868	0.543	0.012	0.430
Fatty acid	Decenoic acid	HMDB0041012	0.205	-0.204	0.654	0.132	-0.196	0.370	0.429	0.049	0.486	0.416	0.483
Organic acid	Decylpropane-tricarboxylic acid		0.000	1.557	1.600	0.000	1.301	0.718	0.000	1.734	0.832	0.000	0.621
Bile acid	Deoxycholic acid	HMDB0000626	0.692	-0.025	0.298	0.892	0.301	0.439	0.879	0.297	0.355	0.959	0.373
Fatty acid	Deoxy-dimethyl-PGE2		0.509	-0.167	0.734	0.877	0.203	0.077	0.290	0.320	0.080	0.006	0.094
Fatty acid	Diaminodecanedioic acid		0.692	0.207	0.936	0.000	0.804	0.511	0.001	0.828	0.433	0.000	0.485
Fatty acid	Diaminododecanedioic acid		0.297	0.281	0.478	0.589	0.079	0.186	0.463	0.225	0.213	0.727	0.462
Fatty acid	Dihomo-γ-linolenic acid	HMDB0002925	0.003	-1.116	1.870	0.012	-1.025	2.106	0.070	-0.751	1.473	0.000	1.647
Bile acid	Dihydroxycholestenoic acid		0.895	0.107	0.750	0.877	-0.014	0.093	0.007	0.497	0.187	0.000	0.212
Fatty acid	Dihydroxy-methyloctanoic acid	HMDB0031557	0.001	0.735	1.369	0.039	0.363	0.273	0.005	0.596	0.303	0.001	0.371
Fatty acid	Dihydroxy-Octadecanedioic acid	HMDB0000782	0.000	1.082	1.555	0.001	0.905	0.215	0.000	1.275	0.268	0.000	0.196
Fatty acid	Dihydroxyoctanoic acid		0.063	0.473	1.008	0.209	0.331	0.170	0.108	0.424	0.180	0.277	0.170
Organic acid	Dihydroxyphenylpropanoate	HMDB0000423	0.002	0.763	0.887	0.743	0.009	0.108	0.195	0.310	0.115	0.000	0.321
Fatty acid	Dihydroxystearate	HMDB0302281	0.644	-0.305	0.545	0.374	-0.136	0.088	0.452	0.063	0.091	0.050	0.114
Amino acid	Dimethylglycine	HMDB0000092	0.063	0.462	0.516	0.279	-0.048	0.121	0.162	0.206	0.086	0.039	0.199
Fatty acid	Docosadienedioic acid		0.068	0.734	1.004	0.011	0.783	0.043	0.000	1.527	0.070	0.000	0.057

Fatty acid	Docosadienoic acid	HMDB0251556	0.588	-0.340	0.721	0.202	-0.456	0.208	0.717	-0.115	0.135	0.086	0.177
Fatty acid	Docosahexaenoic acid	HMDB0002183	0.002	-0.643	1.260	0.000	-0.934	2.871	0.011	-0.420	1.349	0.000	2.069
Fatty acid	Docosapentaenoic acid	HMDB0006528	0.010	-0.743	1.491	0.002	-0.944	1.703	0.052	-0.444	0.903	0.000	1.446
Fatty acid	Docosatrienoic acid	HMDB0002823	0.091	-0.290	0.861	0.047	-0.390	0.214	0.732	-0.012	0.153	0.014	0.239
Fatty acid	Dodecadienoic acid	HMDB0302621	0.544	0.212	0.725	0.862	-0.043	0.186	0.317	0.369	0.361	0.215	0.421
	Dodecadienoic acid isomer		0.653	0.103	0.632	0.524	0.140	0.159	0.045	0.455	0.206	0.011	0.210
Fatty acid	Dodecanedioic acid	HMDB0000623	0.143	-1.430	1.679	0.147	-1.551	0.718	0.164	-1.295	0.571	0.179	0.563
	Dodecanedioic acid isomer-1		0.037	0.626	2.016	0.055	0.513	0.387	0.007	0.831	0.408	0.007	0.331
	Dodecanedioic acid isomer-2		0.216	0.330	1.830	0.245	0.342	0.224	0.065	0.580	0.278	0.069	0.228
	Dodecanedioic acid isomer-3		0.038	0.645	2.015	0.066	0.523	0.275	0.009	0.978	0.366	0.003	0.275
Fatty acid	Dodecatrienoic acid	HMDB0302622	0.235	-0.231	0.407	0.076	-0.342	0.262	0.634	0.120	0.285	0.176	0.498
	Dodecatrienoic acid isomer		0.535	0.373	0.494	0.743	0.049	0.075	0.051	0.695	0.189	0.011	0.265
Fatty acid	Dodecenedioic acid	HMDB0000933	0.000	1.510	1.602	0.000	1.395	0.588	0.000	1.977	0.674	0.000	0.489
Amino acid	Dopaquinone	HMDB0001229	0.874	0.304	0.425	0.183	-0.236	0.070	0.336	0.154	0.050	0.216	0.141
Fatty acid	Eicosadienoic acid	HMDB0005060	0.073	-0.346	0.864	0.025	-0.418	1.046	0.511	-0.075	0.786	0.052	1.025
Fatty acid	Eicosapentaenoic acid	HMDB0001999	0.240	-0.383	0.667	0.086	-0.590	0.843	0.992	0.025	0.316	0.004	1.061

Fatty acid	Eicosenoic acid	HMDB0002231	0.362	-0.203	0.590	0.364	-0.226	0.820	0.932	0.094	0.835	0.305	0.959
Fatty acid	Erucic acid	HMDB0002068	0.421	-0.158	0.725	0.254	-0.157	0.372	0.077	0.066	0.306	0.042	0.405
Organic acid	Erythronic acid	HMDB0000613	0.761	0.523	0.785	0.000	1.701	0.861	0.000	2.411	1.038	0.000	1.001
Carboxylic acid	Formate	HMDB0000142	0.002	-0.633	1.722	0.001	-0.784	1.987	0.006	-0.513	1.221	0.001	1.361
TCA metabolites	Fumaric acid	HMDB0000134	0.355	0.386	0.783	0.032	0.324	0.149	0.010	0.526	0.145	0.023	0.183
Peptide	Glu Arg Pro		0.011	1.050	1.254	0.008	1.200	0.135	0.001	1.608	0.176	0.000	0.124
Peptide	Glu Ile Arg isomer		0.000	2.059	1.153	0.000	3.149	1.006	0.000	3.255	0.846	0.000	0.686
Peptide	Glu Ile Arg-1		0.002	1.334	1.175	0.000	1.705	0.895	0.000	1.918	0.827	0.000	0.637
Peptide	Glu Ile Arg-2		0.001	1.469	1.257	0.000	1.660	0.180	0.000	2.128	0.208	0.000	0.142
Amino acid	Glutamic acid	HMDB0000148	0.002	1.358	0.967	0.076	0.544	0.366	0.019	0.885	0.379	0.001	0.865
Amino acid	Glutamine	HMDB0000641	0.000	1.074	1.381	0.005	0.867	0.518	0.000	1.289	0.614	0.000	0.505
Carboxylic acid	Glutaric acid	HMDB0000661	0.452	-0.068	0.874	0.714	0.089	0.211	0.202	-0.210	0.182	0.322	0.242
Carboxylic acid	Glycerate	HMDB0000139	0.926	-0.025	0.514	0.832	-0.029	0.207	0.101	0.459	0.277	0.003	0.392
Amino acid	Glycine	HMDB0000123	0.006	0.442	0.945	0.000	0.662	0.451	0.000	0.637	0.363	0.000	0.288
Bile acid	Glycochenodeoxycholic acid	HMDB0000637	0.460	-0.374	0.248	0.475	-0.685	0.490	0.732	-0.462	0.283	0.634	0.248

Bile acid	Glycocholic acid	HMDB0000138	0.061	-1.367	0.856	0.127	0.093	0.525	0.121	-1.675	0.883	0.296	1.254
Bile acid	Glycoursodeoxycholic acid	HMDB0000708	0.187	-0.970	1.070	0.602	-0.320	0.527	0.429	-0.653	0.797	0.321	1.017
Peptide	Gly-Thr	HMDB0028851	0.702	0.188	0.564	0.202	-0.198	0.151	0.205	0.005	0.164	0.032	0.223
Eicosanoid	HDoHE or Dihomo-15-deoxy-12,14-PGJ2	HMDB0005079	0.570	-0.214	0.323	0.499	-0.477	0.252	0.505	-0.276	0.198	0.831	0.277
Fatty acid	Heptanoic acid	HMDB0000666	0.021	0.624	0.624	0.224	0.237	0.517	0.000	0.817	0.940	0.000	0.806
Fatty acid	Hexadecatrienoic acid	HMDB0302991	0.731	0.044	0.673	0.817	-0.126	0.199	0.917	0.196	0.261	0.881	0.295
Fatty acid	Hexadecatrienoic acid isomer		0.653	-0.005	0.670	0.279	-0.351	0.189	0.661	0.203	0.204	0.143	0.254
Fatty acid	Hexanoic acid	HMDB0000535	0.162	0.464	0.931	0.908	0.391	0.707	0.073	0.340	0.591	0.060	0.661
	Hexanoic acid isomer		0.021	-1.462	1.298	0.005	-2.052	0.685	0.048	-1.251	0.363	0.002	0.452
Amino acid	Histidine	HMDB0000177	0.544	0.070	0.595	0.772	0.016	0.143	0.167	0.222	0.141	0.099	0.240
Peptide	Homocitrulline	HMDB0000679	0.002	1.241	0.979	0.058	0.481	0.420	0.003	1.003	0.522	0.002	0.963
Peptide	Homocysteine	HMDB0000742	0.895	0.702	1.009	0.406	0.456	0.100	0.682	0.481	0.085	0.354	0.091
Peptide	Homoglutamine	HMDB0245812	0.027	0.555	0.709	0.031	0.351	0.360	0.105	0.483	0.359	0.131	0.374
Phenol	Homovanillic acid	HMDB0000118	0.383	0.223	0.252	0.002	-0.476	0.145	0.128	-0.135	0.056	0.005	0.217
Fatty acid	Hydroperoxy-octadecadienoic acid		0.895	0.438	0.326	0.575	-0.035	0.105	0.170	0.694	0.138	0.029	0.218
Fatty acid	Hydroxy hendecanoic acid	HMDB0340592	0.006	0.753	1.264	0.028	0.593	0.216	0.003	0.900	0.261	0.007	0.205

	Hydroxy hendecanoic acid isomer		0.061	0.691	1.891	0.256	0.507	0.254	0.024	0.836	0.313	0.025	0.236
Fatty acid	Hydroxy hexadecanoic acid	HMDB0006294	0.154	0.543	1.036	0.137	0.522	0.481	0.001	0.920	0.590	0.001	0.524
	Hydroxy hexadecanoic acid isomer-1		0.136	-0.095	0.651	0.032	-0.278	0.235	0.098	0.256	0.182	0.000	0.266
Fatty acid	Hydroxy pelargonic acid		0.004	0.733	1.307	0.020	0.592	0.654	0.002	0.899	0.737	0.004	0.591
	Hydroxy pelargonic acid isomer		0.004	0.650	1.208	0.093	0.435	0.900	0.030	0.679	1.069	0.034	0.898
Fatty acid	Hydroxy tetradecanoic acid	HMDB0061656	0.091	0.631	1.092	0.306	0.540	0.248	0.007	0.867	0.300	0.009	0.256
Fatty acid	Hydroxy tetradecanoic acid isomer	HMDB0010731	0.057	0.607	1.179	0.118	0.543	0.423	0.003	0.829	0.494	0.005	0.415
Fatty acid	Hydroxyadipic acid	HMDB0000321	0.023	2.124	0.735	0.772	0.594	0.310	0.087	1.565	0.529	0.037	0.964
Amino acid	Hydroxy-aspartate	HMDB0032332	0.196	-0.007	1.007	0.549	-0.387	0.298	0.458	-0.288	0.271	0.316	0.313
Hydroxy acid	Hydroxydecanedioic acid	HMDB0000424	0.003	1.003	1.349	0.076	0.526	0.641	0.002	1.011	0.896	0.001	0.801
Hydroxy acid	Hydroxydecanedioic acid isomer-2	HMDB0000350	0.000	0.976	1.456	0.011	0.696	1.612	0.001	0.899	1.672	0.001	1.384
Hydroxy acid	Hydroxydecanoic acid	HMDB0002203	0.006	0.664	1.236	0.001	0.860	0.450	0.001	1.063	0.409	0.000	0.338
Hydroxy acid	Hydroxydecanoic acid-isomer-1	HMDB0033201	0.003	0.679	1.236	0.041	0.468	0.256	0.006	0.789	0.317	0.011	0.255
Hydroxy acid	Hydroxydodecanedioic acid	HMDB0000413	0.000	1.524	1.746	0.000	1.154	3.836	0.000	1.668	4.416	0.000	3.415
	Hydroxydodecanedioic acid isomer		0.000	2.069	1.763	0.000	1.587	2.729	0.000	2.276	3.344	0.000	2.606
Hydroxy acid	Hydroxydodecanoic acid	HMDB0000387	0.010	0.774	1.288	0.022	0.684	0.232	0.001	0.953	0.272	0.005	0.213

Hydroxy acid	Hydroxydodecanoic acid isomer	HMDB0245154	0.028	0.693	1.206	0.109	0.564	0.251	0.003	0.877	0.317	0.005	0.253
Fatty acid	Hydroxy-dodecenoic acid		0.235	0.377	0.869	0.406	-0.079	0.094	0.407	0.376	0.155	0.059	0.171
Amino acid	Hydroxyglutamic acid	HMDB0002273	0.035	-0.233	1.027	0.279	-0.450	0.416	0.098	-0.450	0.372	0.096	0.400
Fatty acid	Hydroxy-hexadecandioic acid		0.000	1.758	1.665	0.000	1.421	1.557	0.000	2.087	1.983	0.000	1.477
Fatty acid	Hydroxyhexanoic acid	HMDB0001624	0.702	-0.086	0.669	0.189	-0.275	0.189	0.856	-0.051	0.146	0.210	0.186
Benzoic acid	Hydroxyhippurate	HMDB0013678	0.452	0.497	0.416	0.743	0.167	0.022	0.682	0.403	0.059	0.375	0.113
Indolyl carboxylic acid	Hydroxyindoleacetate	HMDB0000763	0.468	0.400	0.919	0.428	-0.105	0.036	0.924	0.277	0.053	0.215	0.083
Organic acid	Hydroxy-kynurenine	HMDB0000732	0.003	-0.448	1.113	0.324	0.148	0.734	0.035	0.380	1.682	0.000	2.320
Fatty acid	Hydroxy-methyloctadienoic acid		0.045	0.502	0.421	0.549	-0.375	0.175	0.962	-0.148	0.075	0.002	0.430
Fatty acid	Hydroxynonanedioic acid		0.245	0.334	0.828	0.158	0.421	0.375	0.073	0.519	0.397	0.263	0.337
	Hydroxynonanedioic acid isomer		0.022	0.566	1.161	0.013	0.541	0.445	0.005	0.702	0.494	0.028	0.395
	Hydroxynonanedioic acid-1		0.561	0.291	1.179	0.978	0.087	0.910	0.238	0.299	0.945	0.068	1.079
	Hydroxynonanedioic acid-2		0.174	-0.857	1.339	0.029	-1.259	1.518	0.123	-0.947	1.171	0.048	1.118
	Hydroxynonanedioic acid-3		0.167	0.508	1.775	0.245	0.248	0.389	0.120	0.405	0.459	0.125	0.436
Hydroxy acid	Hydroxynonanoic acid	HMDB0031513	0.005	0.721	1.212	0.015	0.690	0.795	0.003	0.833	0.823	0.018	0.651
Fatty acid	Hydroxyoctadecanoic acid	HMDB0112182	0.335	0.283	0.732	0.271	0.283	0.456	0.031	0.521	0.516	0.058	0.542

Fatty acid	Hydroxyoctadecanoic acid isomer	HMDB0112181	0.028	-0.239	0.756	0.064	-0.332	0.155	0.568	0.063	0.083	0.000	0.169
Fatty acid	Hydroxy-octadecenoic acid	HMDB0000573	0.013	1.343	0.904	0.000	1.484	1.302	0.000	1.734	1.160	0.000	0.885
Fatty acid	Hydroxyoleate	HMDB0034297	0.579	0.220	0.658	0.877	-0.100	0.177	0.313	0.259	0.258	0.548	0.280
Bile acid	Hydroxy-oxo-cholanoic Acid	HMDB0000541	0.761	0.175	0.613	0.714	0.158	0.065	0.402	0.344	0.047	0.284	0.093
	Hydroxy-oxo-cholanoic Acid isomer		0.926	-0.322	0.488	0.499	-0.455	0.124	0.827	-0.169	0.041	0.808	0.096
Bile acid	Hydroxy-oxo-cholestenoate	HMDB0012458	0.009	0.386	1.039	0.671	-0.046	0.114	0.065	0.320	0.232	0.001	0.307
	Hydroxyoxocholestenoic acid or Dioxo-cholestanoic acid		0.316	-0.034	0.470	0.002	-0.504	0.119	0.145	-0.169	0.058	0.001	0.118
Keto acid	Hydroxy-oxoglutarate	HMDB0060466	0.099	-0.423	0.806	0.001	-1.048	0.269	0.021	-0.594	0.146	0.000	0.199
Fatty acid	Hydroxypentadecanedioic acid	HMDB0031885	0.000	1.708	1.554	0.001	1.296	1.080	0.000	2.099	1.504	0.000	1.202
Fatty acid	Hydroxy-pentadecanoic acid	HMDB0340853	0.122	0.702	1.058	0.189	0.646	0.136	0.002	1.049	0.168	0.001	0.145
Peptide	Hydroxyphenylacetylglycine	HMDB0000735	0.075	-0.213	1.038	0.451	-0.270	0.667	0.648	-0.076	0.644	0.012	0.741
Amino acid	Hydroxyproline	HMDB0000725	0.021	-0.537	1.410	0.000	-1.122	1.002	0.000	-0.924	0.741	0.000	0.657
	Hydroxysuberic acid-1	HMDB0000325	0.002	1.123	1.393	0.043	0.680	0.569	0.002	1.007	0.644	0.002	0.567
	Hydroxysuberic acid-2		0.536	0.298	1.661	0.387	-0.151	0.326	0.551	0.196	0.459	0.052	0.445
Fatty acid	Hydroxytetradecanedioic acid	HMDB0000394	0.000	1.344	1.574	0.001	1.044	4.171	0.000	1.555	5.298	0.000	4.077
Fatty acid	Hydroxytetradecatrienoic acid		0.004	1.036	1.157	0.127	0.542	0.225	0.000	1.250	0.422	0.000	0.437

Fatty acid	Hydroxy-tetradecenoic acid		0.937	0.170	0.715	0.254	-0.129	0.101	0.511	0.355	0.141	0.080	0.161
Amino acid	Hydroxytryptophan	HMDB0000472	0.673	0.184	0.440	0.324	-0.125	0.117	0.739	0.191	0.155	0.432	0.193
Fatty acid	Hydroxyundecanedioic acid		0.000	0.922	1.503	0.006	0.646	1.984	0.001	0.927	2.225	0.001	1.841
Fatty acid	Hydroxyvaleric acid	HMDB0000531	0.376	0.208	0.903	0.969	0.075	0.379	0.804	0.116	0.399	0.718	0.496
	Hydroxyvaleric acid isomer-1		0.205	0.183	0.680	0.524	0.020	0.131	0.356	0.241	0.148	0.070	0.225
	Hydroxyvaleric acid isomer-2		0.597	-0.298	0.631	0.475	-0.763	0.503	0.924	-0.445	0.267	0.002	0.364
	Hydroxyvaleric acid isomer-3		0.038	0.423	0.920	0.847	0.007	0.212	0.581	0.152	0.231	0.002	0.409
	Hydroxyvaleric acid isomer-4		0.802	0.089	0.402	0.643	-0.263	0.176	0.634	-0.045	0.188	0.472	0.194
	Hydroxyvaleric acid isomer-5		0.000	1.272	1.394	0.056	0.707	0.466	0.004	0.913	0.543	0.000	0.740
Peptide	Ile Ser	HMDB0028916	0.003	0.961	1.315	0.086	0.484	0.481	0.003	0.957	0.680	0.001	0.617
Indolyl carboxylic acid	Indole-3-acetic acid	HMDB0000197	0.663	0.021	0.431	0.699	-0.100	0.527	0.013	0.340	0.690	0.000	0.890
Carboxylic acid	Isobutyric acid	HMDB0001873	0.316	0.157	0.939	0.954	0.000	0.616	0.150	0.281	0.915	0.036	1.169
Bile acid	Isodeoxycholic acid	HMDB0002536	0.926	0.157	0.313	0.127	-0.391	0.448	0.682	0.311	0.219	0.014	0.468
Amino acid	Isoglutamine	HMDB0003423	0.864	0.161	0.584	0.086	-0.219	0.145	0.036	-0.080	0.150	0.005	0.205
Amino acid	Isoleucine	HMDB0000172	0.003	0.656	1.197	0.086	0.348	0.995	0.014	0.499	1.019	0.001	1.174

Bile acid	Isolithocholic acid	HMDB0000717	0.926	-0.150	0.337	0.364	-1.125	0.096	0.703	-0.251	0.038	0.052	0.096
Fatty acid	Isopropylmalic acid	HMDB0000402	0.405	-2.380	1.608	0.079	-2.786	0.687	0.212	-2.505	0.591	0.045	0.663
Fatty acid	Isovaleric acid	HMDB0000718	0.444	0.307	0.896	0.239	0.351	1.102	0.000	0.929	1.826	0.000	1.718
Peptide	Isowillardiine	HMDB0030376	0.509	0.768	0.546	0.288	0.868	0.102	0.924	1.051	0.096	0.394	0.070
Organic acid	Kynurenine	HMDB0000684	0.008	0.538	0.995	0.122	0.205	0.146	0.008	0.522	0.199	0.003	0.217
Hydroxy acid	Lactic acid	HMDB0000190	0.001	-2.850	1.323	0.000	-2.237	4.076	0.000	-2.307	3.368	0.000	2.962
Fatty acid	Lauric acid	HMDB0000638	0.094	-0.579	0.822	0.082	-0.524	2.065	0.070	-0.463	1.405	0.299	1.493
Peptide	Leu Ser	HMDB0028938	0.001	0.930	1.419	0.015	0.660	1.242	0.001	0.844	1.271	0.003	1.071
Peptide	Leu Val Lys		0.150	-0.243	0.707	0.239	-0.212	0.159	0.261	-0.037	0.157	0.509	0.200
Amino acid	Leucine	HMDB0000687	0.043	0.341	0.753	0.714	0.034	0.705	0.299	0.176	0.741	0.019	1.232
Fatty acid	Lignoceric acid	HMDB0002003	0.895	-0.310	0.707	0.629	-0.561	0.368	0.627	0.028	0.264	0.359	0.297
	Linderic acid or isomer		0.635	0.278	0.707	0.699	-0.183	0.203	0.530	0.342	0.305	0.472	0.300
Fatty acid	Linolenic acid	HMDB0001388	0.916	0.085	0.475	0.602	-0.098	1.344	0.435	0.361	1.832	0.192	2.200
Bile acid	Lithocholic acid	HMDB0000761	0.355	-0.676	0.541	0.231	-1.172	0.117	0.661	-0.556	0.056	0.045	0.086
Bile acid	Lithocholic acid glycine conjugate	HMDB0000698	0.782	-0.945	0.820	0.524	-1.475	0.123	0.909	-0.874	0.080	0.192	0.113
Amino acid	Lysine	HMDB0000182	0.073	0.575	0.951	0.047	0.574	0.570	0.025	0.719	0.574	0.059	0.457

	Lysine or isomer		0.154	0.463	0.751	0.153	0.424	1.329	0.069	0.572	1.394	0.243	1.280
TCA metabolites	Malic acid	HMDB0000744	0.328	-0.004	0.847	0.034	-0.428	0.137	0.098	-0.268	0.113	0.121	0.173
Organic acid	Melanic acid	HMDB0000130	0.027	1.164	0.843	0.026	1.322	4.377	0.000	1.541	3.497	0.002	2.765
Amino acid	Methionine	HMDB0000696	0.229	0.278	0.464	0.451	0.148	0.166	0.125	0.354	0.180	0.356	0.229
Amino acid	Methylene-glutamine		0.000	-1.350	1.572	0.001	-1.476	0.263	0.001	-1.237	0.205	0.001	0.154
Amino acid	Methylhistidine	HMDB0000479	0.979	-0.006	0.283	0.832	-0.102	0.047	0.549	0.104	0.036	0.520	0.087
Fatty acid	Methyl-trihydroxyoctadecenoate	HMDB0302290	0.002	1.069	1.365	0.002	1.016	0.231	0.000	1.407	0.272	0.000	0.204
Fatty acid	Myristic acid	HMDB0000806	0.017	0.232	0.620	0.417	0.100	1.857	0.105	0.254	1.716	0.083	1.747
Benzoic acid	N-Acetylanthranilic acid	HMDB0032388	0.398	0.135	0.280	0.602	-0.236	0.199	0.523	0.312	0.282	0.075	0.557
Peptide	N-Acetyl-aspartate	HMDB0000812	0.214	-0.164	1.000	0.562	-0.334	0.361	0.469	-0.456	0.304	0.409	0.389
Peptide	N-Acetyl-L-citrulline	HMDB0000856	0.303	0.295	0.484	0.463	-0.151	0.237	0.675	0.144	0.292	0.034	0.393
Peptide	N-Acetyl-ornithine	HMDB0242109	0.452	0.169	0.599	0.671	-0.021	0.098	0.962	0.174	0.145	0.584	0.163
Peptide	N-carbamyl-L-glutamate	HMDB0015673	0.297	0.003	1.006	0.892	-0.179	0.469	0.648	-0.137	0.411	0.510	0.429
Fatty acid	Nervonic acid	HMDB0002368	0.460	-0.054	0.404	0.279	0.062	0.237	0.040	0.244	0.254	0.011	0.311
Peptide	N-lactoyl-phenylalanine	HMDB0062175	0.006	1.304	1.001	0.079	0.618	0.441	0.002	1.230	0.592	0.004	0.938
Fatty acid	Nonadecanoic acid	HMDB0000772	0.170	0.511	1.178	0.209	0.427	0.975	0.068	0.572	1.021	0.284	0.817

Fatty acid	Nonanedioic acid	HMDB0000784	0.002	1.130	1.377	0.001	1.195	4.008	0.000	1.420	3.650	0.001	2.872
Fatty acid	Nonylenic acid	HMDB0031271	0.009	0.672	1.169	0.013	0.629	0.452	0.002	0.838	0.497	0.008	0.409
	Nonylenic acid isomer-1		0.122	0.186	0.550	0.019	0.409	0.128	0.002	0.524	0.179	0.003	0.234
	Nonylenic acid isomer-2		0.027	0.626	0.666	0.045	0.696	0.304	0.003	0.878	0.307	0.017	0.285
Fatty acid	Octanoic acid	HMDB0000482	0.721	0.443	0.362	0.985	0.594	0.863	0.227	0.204	0.647	0.487	1.246
	Octanoic acid isomer		0.010	1.647	0.492	0.024	-0.531	0.236	0.308	1.567	0.905	0.006	1.268
Peptide	Octanoyl-aspartic acid		0.012	1.338	1.096	0.079	1.020	0.388	0.007	1.201	0.291	0.042	0.310
Fatty acid	Octenedioic acid	HMDB0004982	0.005	0.769	1.339	0.109	0.387	0.590	0.034	0.541	0.639	0.005	0.695
Fatty acid	Octenoic acid	HMDB0000392	0.822	0.074	0.611	0.170	-0.083	0.221	0.753	0.204	0.364	0.229	0.476
Fatty acid	Oleic acid	HMDB0000207	0.895	-0.078	0.708	0.589	0.039	4.243	0.195	0.195	4.585	0.047	4.952
Amino acid	Ornithine	HMDB0000214	0.625	-0.009	0.405	0.093	-0.356	0.084	0.145	-0.183	0.025	0.173	0.095
Keto acid	Oxoadipate	HMDB0000225	0.428	-3.104	1.385	0.728	-2.593	0.891	0.530	-2.201	0.663	0.000	0.657
Fatty acid	Oxo-decanedioic acid		0.017	0.758	0.638	0.031	0.622	0.234	0.001	1.268	0.295	0.000	0.279
Keto acid	Oxooctanoic acid	HMDB0010721	0.000	1.204	1.375	0.000	0.964	0.224	0.000	1.412	0.259	0.000	0.190
	Oxooctanoic acid-2		0.856	0.044	0.772	1.000	0.027	0.163	0.259	0.333	0.279	0.196	0.264
Fatty acid	Oxosuberate		0.007	0.864	1.175	0.189	0.357	0.165	0.013	0.678	0.201	0.002	0.235

Keto acid	Oxosuccinamate	HMDB0060350	0.297	-0.187	0.553	0.006	-0.648	0.068	0.052	-0.421	0.043	0.009	0.069
Fatty acid	Oxo-tetradecenoic acid		0.509	0.176	0.711	0.306	-0.111	0.093	0.668	0.275	0.136	0.066	0.153
Fatty acid	Oxotridecadienoic acid		0.024	0.805	0.620	0.176	0.375	0.078	0.004	1.001	0.200	0.001	0.171
Fatty acid	Oxo-undecanedioic acid		0.004	1.005	1.393	0.051	0.645	0.333	0.004	0.911	0.360	0.006	0.332
Fatty acid	Palmitelaidic acid	HMDB0012328	0.905	0.085	0.581	0.615	0.116	2.164	0.775	0.181	2.367	0.958	2.082
Fatty acid	Palmitic acid	HMDB0000220	0.444	0.059	0.676	0.086	0.132	3.419	0.023	0.296	4.102	0.002	4.145
Fatty acid	Pelargonic acid	HMDB0000847	0.187	0.369	0.984	0.022	0.523	2.380	0.003	0.708	2.510	0.002	2.063
Fatty acid	Pentadecadienoic acid	HMDB0302695	0.122	-0.160	0.855	0.297	-0.109	0.531	0.245	0.232	0.615	0.001	0.781
Fatty acid	Pentadecanoic acid	HMDB0000826	0.119	0.241	0.624	0.239	0.227	0.629	0.043	0.371	0.656	0.148	0.660
Eicosanoid	PGF1a	HMDB0002685	0.075	0.581	1.275	0.562	0.065	0.087	0.594	0.016	0.056	0.000	0.171
Eicosanoid	PGF1a isomer		0.000	0.878	1.770	0.009	0.736	0.117	0.030	0.642	0.091	0.000	0.090
Amino acid	Phenylalanine	HMDB0000159	0.444	0.097	0.652	0.022	-0.306	1.042	0.327	-0.097	0.675	0.001	1.436
Fatty acid	Phenyl-trihydroxy-hexanoic acid		0.187	0.459	0.703	0.602	-0.292	0.262	0.261	0.425	0.523	0.004	1.080
Organic acid	Picolinic acid	HMDB0002243	0.068	0.231	0.858	0.847	-0.062	0.128	0.070	0.283	0.196	0.002	0.257
Fatty acid	Pimelic acid	HMDB0000857	0.187	-0.329	1.008	0.271	-0.267	0.540	0.381	-0.283	0.420	0.585	0.419

Peptide	Pipecolic acid	HMDB0000070	0.291	0.093	0.236	0.045	0.207	0.859	0.027	0.297	1.065	0.006	1.069
Peptide	Piperidine-2,6-dicarboxylate	HMDB0012289	0.116	-0.319	0.996	0.406	-0.243	0.222	0.710	0.036	0.267	0.067	0.369
Amino acid	Proline	HMDB0000162	0.008	0.865	1.025	0.025	0.751	0.585	0.004	0.936	0.579	0.024	0.526
Amino acid	Propenylproline		0.625	0.263	0.472	0.324	-0.319	0.449	0.668	0.045	0.354	0.176	0.702
Carboxylic acid	Propionic acid	HMDB0000237	0.452	-0.076	0.718	0.029	-0.297	0.653	0.176	-0.147	0.439	0.071	0.705
Amino acid	Pyroglutamic acid	HMDB0000267	0.002	-0.793	1.762	0.002	-0.800	1.257	0.007	-0.651	0.866	0.006	0.902
Keto acid	Pyruvic acid	HMDB0000243	0.235	-0.916	1.284	0.089	-1.255	0.296	0.269	-0.878	0.195	0.106	0.241
Amino acid	Sarcosine	HMDB0000271	0.235	0.143	0.629	0.011	0.431	0.617	0.090	0.210	0.353	0.038	0.480
Peptide	S-Carboxymethyl-cysteine	HMDB0029415	0.019	-0.372	0.972	0.288	-0.309	0.085	0.265	-0.335	0.095	0.009	0.104
Fatty acid	Sebacic acid	HMDB0000792	0.653	-0.041	0.993	0.892	0.013	0.987	0.424	0.271	0.872	0.084	0.857
	Sebacic acid isomer		0.692	-1.792	1.709	0.817	-1.872	2.618	0.804	-1.426	2.009	0.229	2.168
Amino acid	Serine	HMDB0062263	0.102	-1.439	0.601	0.153	-1.025	0.253	0.105	-1.116	0.243	0.231	0.275
Fatty acid	Stearic acid	HMDB0000827	0.196	0.094	0.586	0.037	0.145	2.902	0.008	0.335	3.841	0.000	3.716
Fatty acid	Suberic acid	HMDB0000893	0.019	0.720	1.210	0.013	0.693	0.541	0.005	0.829	0.537	0.029	0.431
	Suberic acid-2		0.712	-0.007	0.985	0.892	0.039	0.955	0.555	0.238	0.809	0.314	0.806
TCA metabolites	Succinate	HMDB0000254	0.291	0.043	0.931	0.118	-0.346	0.243	0.977	0.007	0.215	0.017	0.268

Fatty acid	Tetradecadienoic acid		0.926	0.074	0.653	0.728	-0.215	0.261	0.819	0.272	0.398	0.617	0.435
Fatty acid	Tetradecanedioic acid	HMDB0000872	0.002	0.928	1.293	0.001	0.914	0.761	0.000	1.184	0.775	0.000	0.562
Fatty acid	Tetradecatrienoic acid		0.245	0.060	0.566	0.728	-0.168	0.073	0.614	0.063	0.073	0.088	0.095
Fatty acid	Tetradecenoic acid	HMDB0302697	0.034	0.778	0.890	0.189	0.747	0.862	0.156	0.777	0.747	0.293	0.560
	Tetradecenoic acid isomer-1		0.073	0.441	0.705	0.246	0.114	0.365	0.027	0.655	0.518	0.111	0.458
	Tetradecenoic acid isomer-2		0.968	0.328	0.758	0.142	-0.165	0.175	0.648	0.474	0.290	0.126	0.343
Fatty acid	Tetrahydroxyoctadecadienoic acid		0.154	0.837	1.020	0.006	1.165	0.262	0.002	1.355	0.226	0.000	0.184
Amino acid	Threonine	HMDB0000167	0.002	1.032	1.201	0.005	0.912	0.360	0.001	1.167	0.352	0.003	0.287
Benzoic acid	Tolylacetic acid	HMDB0002222	0.509	-0.280	0.501	0.832	-1.057	0.415	0.238	-0.418	0.236	0.003	0.414
Fatty acid	Tridecanoic acid	HMDB0000910	0.003	0.584	1.129	0.003	0.586	0.421	0.000	0.764	0.450	0.000	0.345
	Tridecanoic acid isomer		0.070	0.273	0.913	0.043	0.292	0.503	0.001	0.540	0.615	0.000	0.505
Fatty acid	Tridecenoic acid		0.182	-0.194	0.673	0.757	0.058	0.161	0.164	0.401	0.255	0.000	0.306
Fatty acid	Trihydroxy-octadecenoic acid	HMDB0030936	0.001	1.127	1.388	0.002	1.065	0.554	0.000	1.426	0.634	0.000	0.465
Fatty acid	Trimethyldodecatrienoic acid		0.009	0.906	0.746	0.053	0.546	0.253	0.014	0.644	0.251	0.026	0.250
Amino acid	Tryptophan	HMDB0000929	0.096	0.276	0.874	0.802	0.024	0.478	0.041	0.308	0.859	0.015	1.028
Eicosanoid	TXB2	HMDB0003252	0.792	0.071	0.488	0.757	0.139	0.091	0.561	0.532	0.138	0.270	0.158
Peptide	Tyr-Asp-OH		0.086	0.869	0.954	0.908	-0.192	0.089	0.002	1.424	0.235	0.000	0.237

Amino acid	Tyrosine	HMDB0000158	0.802	-0.246	0.758	0.772	-0.184	0.463	0.607	-0.069	0.401	0.200	0.622
Fatty acid	Undecanedicarboxylic acid	HMDB0002327	0.002	1.226	1.238	0.035	0.823	0.353	0.002	1.317	0.430	0.006	0.401
	Undecanedicarboxylic acid isomer-1		0.003	0.838	1.246	0.002	0.773	0.468	0.001	1.041	0.495	0.002	0.365
	Undecanedicarboxylic acid isomer-2		0.000	2.279	1.543	0.000	1.928	0.529	0.000	2.424	0.531	0.000	0.444
Fatty acid	Undecanedioic acid	HMDB0000888	0.905	0.247	1.011	0.643	0.345	1.051	0.083	0.630	0.947	0.036	0.904
Fatty acid	Undecanoic acid	HMDB0000947	0.003	0.917	1.231	0.001	0.913	0.793	0.000	1.216	0.842	0.000	0.595
Fatty acid	Undecenoic acid	HMDB0033724	0.383	0.342	0.745	0.728	-0.105	0.240	0.189	0.383	0.391	0.051	0.538
Bile acid	Ursodeoxycholic acid	HMDB0000946	0.224	0.151	0.113	0.395	0.111	0.425	0.030	0.538	0.407	0.019	0.504
Peptide	Val Ile Leu		0.056	-0.370	0.820	0.297	-0.180	0.099	0.090	-0.309	0.141	0.158	0.148
Fatty acid	Valeric acid	HMDB0000892	0.405	0.281	1.001	0.082	0.334	1.797	0.000	0.955	3.335	0.000	3.032
Amino acid	Valine	HMDB0000883	0.605	0.031	0.390	0.148	-0.368	0.322	0.382	-0.161	0.283	0.000	0.341
	Valine or isomer		0.019	0.760	0.687	0.475	-0.263	0.362	0.130	0.270	0.372	0.000	0.583
Fatty acid ester	Valinopine or Malonylcarnitine	HMDB0002095	0.328	-0.240	0.435	0.122	-0.126	0.135	0.017	0.705	0.188	0.001	0.231
Benzoic acid	Vanilloylglycine	HMDB0060026	0.214	-0.369	1.032	0.685	-0.446	0.439	0.530	-0.329	0.464	0.101	0.498
	(2S)-2,5-diamino-5-oxopentanoic acid; phosphane		0.771	0.085	0.941	0.387	0.142	0.984	0.023	0.544	5.814	0.000	3.902

	Unidentified1		0.014	0.930	1.085	0.001	1.547	1.370	0.000	1.828	1.884	0.000	1.619
	Unidentified2		0.005	1.046	1.240	0.000	2.033	1.818	0.000	2.375	1.745	0.000	1.518
Benzenoids	4-O-Methylgallic acid	HMDB0013198	0.106	0.697	0.643	0.490	0.478	0.427	0.065	0.910	1.205	0.055	1.561
Organic acids	N-Myristoyl Lysine	HMDB0242055	0.066	0.634	1.114	0.062	0.495	1.124	0.046	0.689	2.071	0.123	2.124
Organic acids	3-hydroxy-2-octyl-glutaric acid		0.000	2.242	1.542	0.002	1.759	1.108	0.000	2.515	2.298	0.000	2.190
Organic acids	Dibutyl malate	HMDB0031696	0.007	1.412	1.308	0.047	0.782	0.770	0.001	1.997	2.239	0.000	1.941
	Glycerolipids		0.002	2.018	1.365	0.010	1.484	0.999	0.000	2.630	2.572	0.000	2.145
Glyceride	Diglyceride		0.032	1.082	1.115	0.001	1.304	1.019	0.002	1.512	1.695	0.001	1.569
	Unidentified3		0.330	-2.765	1.609	0.907	-8.677	1.657	0.384	-9.471	3.660	0.004	2.414
	Unidentified4		0.000	2.175	1.485	0.003	1.784	1.065	0.000	2.525	1.976	0.000	1.921
	Unidentified5		0.000	1.734	1.586	0.001	1.516	1.186	0.000	2.131	2.983	0.000	2.615
Lipids	LysoPC(18:3(9Z,12Z,15Z)/0:0)	HMDB0010388	0.484	-0.086	0.972	0.000	-1.182	1.427	0.064	-0.295	1.039	0.000	1.833
Lipids	LysoPC(16:1(9Z)/0:0)	HMDB0010383	0.507	0.228	0.987	0.746	0.091	0.804	0.016	0.809	2.528	0.000	1.843
Lipids	LysoPC(18:2(9Z,12Z)/0:0)	HMDB0010386	0.029	-0.490	1.307	0.009	-0.581	1.329	0.640	-0.044	7.115	0.000	6.725
Lipids	LysoPC(20:4(5Z,8Z,11Z,14Z)/0:0)	HMDB0010395	0.252	-0.202	0.997	0.534	-0.127	0.585	0.092	0.446	4.983	0.000	4.186
Lipids	LysoPC(22:6(4Z,7Z,10Z,13Z,16Z,19Z)/0:0)	HMDB0010404	0.040	-0.595	1.272	0.031	-0.611	1.081	0.785	-0.038	2.423	0.000	2.530
Lipids	LysoPC(22:5(4Z,7Z,10Z,13Z,16Z)/0:0)	HMDB0010402	0.313	-0.259	0.983	0.705	-0.133	0.622	0.132	0.497	2.025	0.000	1.807

Lipids	PC(16:0/22:4(7Z,10Z,13Z,16Z)) or isomer	HMDB0007988	0.000	2.331	1.014	0.000	2.299	0.977	0.000	3.583	2.744	0.000	1.822
Lipids	LysoPC(16:0/0:0)	HMDB0010382	0.173	-0.321	1.191	0.234	-0.325	1.119	0.979	0.049	8.619	0.002	6.448
Lipids	LysoPC(20:3(8Z,11Z,14Z)/0:0)	HMDB0010394	0.611	0.031	0.909	0.441	0.239	0.799	0.017	0.693	3.164	0.000	2.260
Lipids	LysoPC(18:1(9Z)/0:0)	HMDB0002815	0.102	-0.353	1.228	0.074	-0.364	1.094	0.938	0.087	6.662	0.000	5.392
Lipids	LysoPC(17:0/0:0)	HMDB0012108	0.161	-0.376	1.103	0.074	-0.590	1.138	0.508	-0.082	1.969	0.001	1.862
Benzenoids	Bis(2-ethylhexyl) phthalate	HMDB0249243	0.000	-1.792	2.283	0.000	-2.245	1.752	0.000	-2.106	2.527	0.000	2.018
Sphingolipids	SM(d18:1/16:1(9Z))	HMDB0240613	0.260	-0.421	0.802	0.204	-0.568	0.845	0.330	-0.341	2.974	0.465	2.439
Lipids	PC(14:0/20:2(11Z,14Z)) or isomer	HMDB0007880	0.192	1.326	0.598	0.286	-1.344	0.541	0.003	2.400	30.392	0.000	25.196
Lipids	PC(16:0/22:6(4Z,7Z,10Z,13Z,16Z,19Z)) or isomer	HMDB0007991	0.001	2.674	1.210	0.003	2.373	1.051	0.000	2.683	10.801	0.005	11.136
Lipids	LysoPC(18:0/0:0)	HMDB0010384	0.003	-0.642	1.720	0.001	-0.780	1.532	0.033	-0.421	10.012	0.002	8.605
	Unidentified6		0.343	2.921	0.441	0.414	1.920	0.961	0.012	0.437	0.525	0.027	2.112
Lipids	LysoPG(16:0/0:0)	HMDB0240601	0.001	-2.672	1.542	0.047	-1.544	0.694	0.005	-2.322	1.677	0.001	1.805
Fatty amides	Fatty amides		0.000	-1.237	1.997	0.123	-0.332	0.417	0.008	-0.688	0.989	0.000	1.814
	Unidentified7		0.003	0.623	0.355	0.002	-0.059	0.999	0.000	-0.754	0.637	0.000	1.670
Lipids	LysoPC(18:0/0:0) isomer	HMDB0010384	0.000	-0.813	2.086	0.000	-1.326	2.002	0.000	-1.163	2.235	0.000	1.775
Lipids	LysoPC(20:1(11Z)/0:0)	HMDB0010391	0.000	-1.133	2.229	0.000	-1.671	1.929	0.001	-1.060	1.970	0.000	1.772

Fatty amides	13-Docosenamide	HMDB0244507	0.005	-0.857	1.368	0.003	-1.107	1.256	0.022	-0.740	7.287	0.026	6.904
Benzoic acids	Didecyl phthalate or isomer	HMDB0251217	0.000	-2.422	2.091	0.000	-5.504	1.787	0.000	-2.581	1.971	0.000	1.772
	1-Palmitoyl-2-(9-oxo-nonanoyl)-sn-glycero-3-phosphocholine		0.087	-0.204	0.647	0.004	-1.035	0.958	0.385	-0.100	0.984	0.000	1.697
	Unidentified8		0.001	-1.429	1.664	0.000	-8.878	2.078	0.000	-9.612	3.184	0.000	2.360
Lipids	SM(d16:1/20:5(7Z,9Z,11E,13E,17Z)-3OH(5,6,15))	HMDB0290285	0.000	2.351	1.189	0.001	2.055	1.100	0.000	2.766	4.348	0.000	2.922
Alcohols and polyols	(2S,3R)-2-Amino-4-octadecene-3-ol	HMDB0242695	0.072	-0.690	0.835	0.070	0.791	0.642	0.702	0.419	0.967	0.000	1.539
Sphingolipids	SM(d18:1/16:0)	HMDB0010169	0.326	-0.957	0.878	0.766	-0.469	0.278	0.436	-0.793	1.495	0.520	1.834
Glycerolipids	MG(16:0/0:0/0:0)	HMDB0011564	0.617	0.396	0.765	0.001	0.879	1.336	0.000	1.492	1.632	0.000	1.507
	Vitamin d derivative		0.870	-0.110	0.655	0.552	-0.215	0.647	0.866	0.179	4.286	0.152	4.289
	Unidentified9		0.210	-0.983	1.053	0.018	-1.980	1.229	0.086	-1.282	1.773	0.002	1.539

TABLE S4 | Pearson's correlation analysis between altered metabolites and IgE levels

Class	Name	*Der p* sIgE (KU/L)		*Der f* sIgE (KU/L)		TIgE (KU/L)	
		Correlation coefficient	*P*-Value	Correlation coefficient	*P*-Value	Correlation coefficient	*P*-Value
Fatty acid	13(S)-HPODE	-0.074	0.037	-0.255	0.009	-0.054	0.324
Fatty acid	Eicosapentaenoic acid	0.047	0.516	-0.211	0.032	0.030	0.471
Amino acid	Isoleucine	-0.074	0.312	-0.200	0.043	-0.061	0.409
Amino acid	Phenylalanine	-0.066	0.362	-0.232	0.018	-0.017	0.816
Gamma-keto acid	a-ketoglutaric acid	0.058	0.424	-0.005	0.958	0.153	0.039
Organic acid	Picolinic acid	-0.087	0.232	-0.232	0.018	-0.018	0.812
Short-chain keto acid	Acetoacetate	0.134	0.066	0.075	0.451	0.153	0.038
Amino acid	Dimethylglycine	0.031	0.669	-0.029	0.769	0.310	0.000
Keto acid	2-Oxoglutaramate	0.128	0.078	0.120	0.227	0.215	0.003
Peptide/Amino acids	Pipecolic acid	0.153	0.035	0.041	0.678	0.198	0.007
Peptide/Amino acids	N-Acetyl-L-citrulline	0.091	0.214	0.107	0.282	0.176	0.017
Amino acid	Hydroxyproline	-0.043	0.559	-0.242	0.014	0.053	0.478
Keto acid	Hydroxy oxoglutarate	0.043	0.554	-0.032	0.746	0.158	0.033
TCA metabolites	Fumaric acid	-0.032	0.662	-0.200	0.043	0.067	0.365

Hydroxy acid	Hydroxydecanoic acid	0.089	0.223	-0.080	0.423	0.149	0.044
Bile acid	Cholic acid	0.018	0.810	-0.207	0.036	-0.028	0.710
Peptide	Gly-Thr	0.104	0.152	0.058	0.558	0.182	0.014
Fatty acid	Decadienoic acid	0.073	0.317	-0.013	0.898	0.383	0.000
Bile acid	Dihydroxycholestenoic acid	0.086	0.238	-0.058	0.557	0.165	0.026
Fatty acid	Docosadienedioic acid	-0.115	0.115	-0.259	0.008	0.039	0.603
Bile acid	Hydroxy oxocholestenoate	0.020	0.785	0.023	0.820	0.193	0.009
Organic acid	Hydroxykynurenine	0.127	0.081	-0.064	0.521	0.173	0.020
Fatty acid	Stearic acid	0.145	0.046	0.065	0.514	0.142	0.055
Indolyl carboxylic acid	Indole-3-acetic acid	-0.029	0.689	-0.201	0.042	-0.025	0.734
Hydroxy acid	Lactic acid	0.133	0.067	0.061	0.543	0.201	0.006
Organic acid	Melanic acid	0.271	0.000	0.146	0.140	0.232	0.002
Benzenoids	4-O-Methylgallic acid	0.258	0.000	0.085	0.392	0.146	0.049
Lipids	LysoPC(16:1(9Z)/0:0)	0.110	0.131	-0.031	0.760	0.157	0.034
Lipids	PC(16:0/22:4)	0.147	0.042	-0.059	0.553	0.157	0.034
Lipids	PC(14:0/20:2)	0.149	0.040	0.066	0.506	0.258	0.000
Lipids	PC(16:0/22:6)	-0.052	0.480	-0.208	0.035	0.006	0.935

Glycerophospholipids	PoxnoPC	0.152	0.037	-0.003	0.978	0.117	0.115
Glycerolipids	MG(16:0/0:0/0:0)	0.101	0.166	0.018	0.855	0.164	0.027
Amino acid	Methylene glutamine	0.124	0.043	-0.064	0.519	0.179	0.027
Fatty acid ester	Acetylcarnitine	0.128	0.077	-0.062	0.536	0.140	0.021
Fatty acid	Aminoheptanoic acid	0.106	0.144	0.001	0.989	0.163	0.015

TABLE S5 | Diagnostic performance of specific metabolites for each AADs

	Metabolites	AUC	95% CI	Sensitivity	Specificity	Cutoffs
	Aminophenyl-dioxobutanoic acid	0.836	0.725-0.946	0.714	0.810	12.524
	Dihydroxyphenylpropanoate	0.761	0.634-0.888	0.581	0.929	0.224
	Hydroxy-oxo-cholestenoate	0.726	0.581-0.870	0.730	0.786	0.395
	Benzoic acid	0.723	0.564-0.883	1.000	0.429	0.657
	Acetyllysine	0.721	0.571-0.871	0.746	0.714	3.790
AR	Myristic acid	0.704	0.523-0.885	0.905	0.571	16.813
	Allylglycine	0.703	0.541-0.865	0.730	0.714	1.066
	Valine or isomer	0.702	0.517-0.886	0.794	0.714	0.750
	S-Carboxymethyl-cysteine	0.701	0.554-0.847	0.571	0.825	0.051
	Cyano-aminobutyric acid	0.697	0.517-0.877	0.984	0.413	0.014
	Hydroxyadipic acid	0.695	0.552-0.838	0.683	0.786	0.271

	Arginine	0.693	0.540-0.846	0.841	0.500	0.080
	Carbamyl-aspartate	0.689	0.538-0.840	0.929	0.429	0.049
	Hydroxyoctadecanoic acid isomer	0.688	0.545-0.831	0.786	0.619	0.154
	Tetradecenoic acid	0.683	0.531-0.834	0.730	0.643	1.068
	Hydroxyglutamic acid	0.681	0.520-0.842	0.500	0.905	0.623
	3-dodecenoic acid or isomer	0.679	0.511-0.848	0.952	0.429	0.123
	Hydroxyvaleric acid isomer	0.678	0.514-0.842	0.619	0.714	0.683
	Alanylleucine isomer	0.677	0.495-0.858	0.825	0.539	1.622
	Leucine	0.673	0.516-0.831	0.841	0.571	5.103
	Amino-acetoacetate	0.672	0.483-0.862	0.921	0.500	0.148
	Hydroxy-kynurenine	0.752	0.627-0.876	0.786	0.730	12.750
	1-(Hydroxymethyl)-5-oxo-L-proline	0.672	0.513-0.831	0.786	0.667	7.388
	Hydroxy-methyloctadienoic acid	0.672	0.508-0.837	0.619	0.786	0.276
	LysoPC(18:3(9Z,12Z,15Z)/0:0)	0.924	0.843-1.000	0.875	0.878	0.014
	2-Oxo-isocaproate isomer	0.836	0.732-0.940	0.929	0.732	0.521
	Amino-hydroxydodecanoic acid	0.791	0.616-0.966	0.714	0.976	1.423
	Homovanillic acid	0.775	0.634-0.916	0.929	0.585	0.083
Asthma	Oxosuccinamate	0.747	0.600-0.895	0.714	0.780	0.017
	Sarcosine	0.730	0.589-0.871	0.659	0.786	2.075
	Phenylalanine	0.707	0.556-0.859	0.929	0.512	7.022
	Eicosadienoic acid	0.702	0.548-0.856	0.857	0.537	4.252
	3-Indolepropionic acid	0.697	0.519-0.875	0.786	0.707	1.673

Propionic acid	0.697	0.522-0.871	0.571	0.854	3.035
Hydroxy hexadecanoic acid isomer	0.693	0.533-0.854	0.643	0.756	0.404
Malic acid	0.692	0.504-0.880	0.714	0.805	0.094
Docosatrienoic acid	0.679	0.514-0.845	0.643	0.683	0.280
Cholic acid	0.507	0.310-0.696	0.571	0.634	0.117
Octanoic acid isomer	0.704	0.537-0.870	0.571	0.902	0.366
Hydroxyoxocholestenoic acid or Dioxo-cholestanoic acid	0.774	0.643-0.904	0.929	0.659	0.059
Unidentified9	0.767	0.531-1.000	0.625	0.902	0.021
Valeric acid	0.871	0.798-0.944	0.722	1.000	24.282
Cholestenoic acid	0.806	0.711-0.901	0.533	1.000	0.943
Isovaleric acid	0.805	0.677-0.933	0.722	0.857	8.188
Hydroxy hexadecanoic acid	0.770	0.667-0.873	0.622	0.929	1.198
Anthranilic acid	0.765	0.609-0.921	0.744	0.786	0.145
Hydroxy-pentadecanoic acid	0.763	0.663-0.862	0.600	0.929	0.093
CARASPC(14:0/20:2(11Z,14Z)) or isomer	0.815	0.683-0.948	0.811	0.750	0.032
Hydroxy tetradecanoic acid isomer	0.744	0.641-0.848	0.589	0.929	0.885
Hydroxy tetradecanoic acid	0.727	0.618-0.836	0.533	0.929	0.371
Indole-3-acetic acid	0.706	0.554-0.859	0.756	0.643	4.106
LysoPC(16:1(9Z)/0:0)	0.760	0.587-0.933	0.622	0.875	0.063
LysoPC(20:3(8Z,11Z,14Z)/0:0)	0.756	0.622-0.889	0.478	1.000	0.138
Valinopine or Malonylcarnitine	0.698	0.503-0.894	0.978	0.571	0.003

Palmitic acid	0.689	0.537-0.840	0.822	0.571	96.784
Hydroxy hendecanoic acid isomer	0.742	0.596-0.887	0.700	0.750	0.143
Tetradecenoic acid isomer	0.685	0.511-0.858	0.778	0.643	0.483
Decanoic acid	0.681	0.531-0.831	0.644	0.786	4.539
Hydroxyoctadecanoic acid	0.680	0.525-0.835	0.656	0.714	1.342
Behenic acid	0.677	0.501-0.852	0.822	0.571	0.370
Isoglutamine	0.675	0.554-0.795	0.786	0.622	0.185
Nervonic acid	0.671	0.486-0.857	0.856	0.571	0.464
Tryptophan	0.671	0.523-0.818	0.756	0.571	5.495
Pentadecanoic acid	0.669	0.530-0.808	0.478	0.857	3.435
Dodecadienoic acid isomer	0.667	0.538-0.796	0.522	0.857	0.235
N-Myristoyl Lysine	0.715	0.532-0.897	0.822	0.625	0.030
Tyr-Asp-OH	0.761	0.639-0.883	0.444	1.000	0.134
Dihydroxycholestenoic acid	0.724	0.598-0.850	0.522	0.857	0.226
2-[2-ethoxy-4-(hydroxymethyl)phenoxy]pentanoic acid	0.719	0.570-0.868	0.789	0.571	0.699
(2S)-2,5-diamino-5-oxopentanoic acid; phosphane	0.743	0.581-0.905	0.922	0.500	0.295
Ursodeoxycholic acid	0.681	0.510-0.852	0.633	0.786	1.330
Unidentified6	0.697	0.50-0.893	0.625	0.811	0.015

TABLE S6 | Age-adjusted comparison of differential metabolites between disease groups and healthy using ANCOVA

Metabolite	Comparison (Group vs. Healthy)	Raw P-value	Adjusted P-value (ANCOVA)	Significant after adjustment
LysoPC(18:3(9Z,12Z,15Z)/0:0)	Asthma vs. Healthy	0.0002	0.0023	Yes
Sarcosine	Asthma vs. Healthy	0.0108	0.0094	Yes
Phenylalanine	Asthma vs. Healthy	0.0215	0.0381	Yes
Eicosadienoic acid	Asthma vs. Healthy	0.0250	0.0156	Yes
Propionic acid	Asthma vs. Healthy	0.0290	0.0266	Yes
Docosatrienoic acid	Asthma vs. Healthy	0.0466	0.0482	Yes
Aminophenyl-dioxobutanoic acid	AR vs. Healthy	0.0001	0.0018	Yes
Dihydroxyphenylpropanoate	AR vs. Healthy	0.0024	0.0018	Yes
Benzoic acid	AR vs. Healthy	0.0093	0.0145	Yes
Hydroxy-kynurenine	AR vs. Healthy	0.0034	0.0164	Yes
Valeric acid	CARAS vs. Healthy	0.0000	0.0004	Yes
Cholestenoic acid	CARAS vs. Healthy	0.0002	0.0000	Yes
Indole-3-acetic acid	CARAS vs. Healthy	0.0133	0.0274	Yes
Hydroxypentadecanedioic acid	CARAS vs. Healthy	0.0000	0.0004	Yes
Isovaleric acid	CARAS vs. Healthy	0.0003	0.0045	Yes
Hydroxy-hexadecandioic acid	CARAS vs. Healthy	0.0000	0.0010	Yes
LysoPC(16:1(9Z)/0:0)	CARAS vs. Healthy	0.0155	0.0374	Yes
Palmitic acid	CARAS vs. Healthy	0.0234	0.0208	Yes

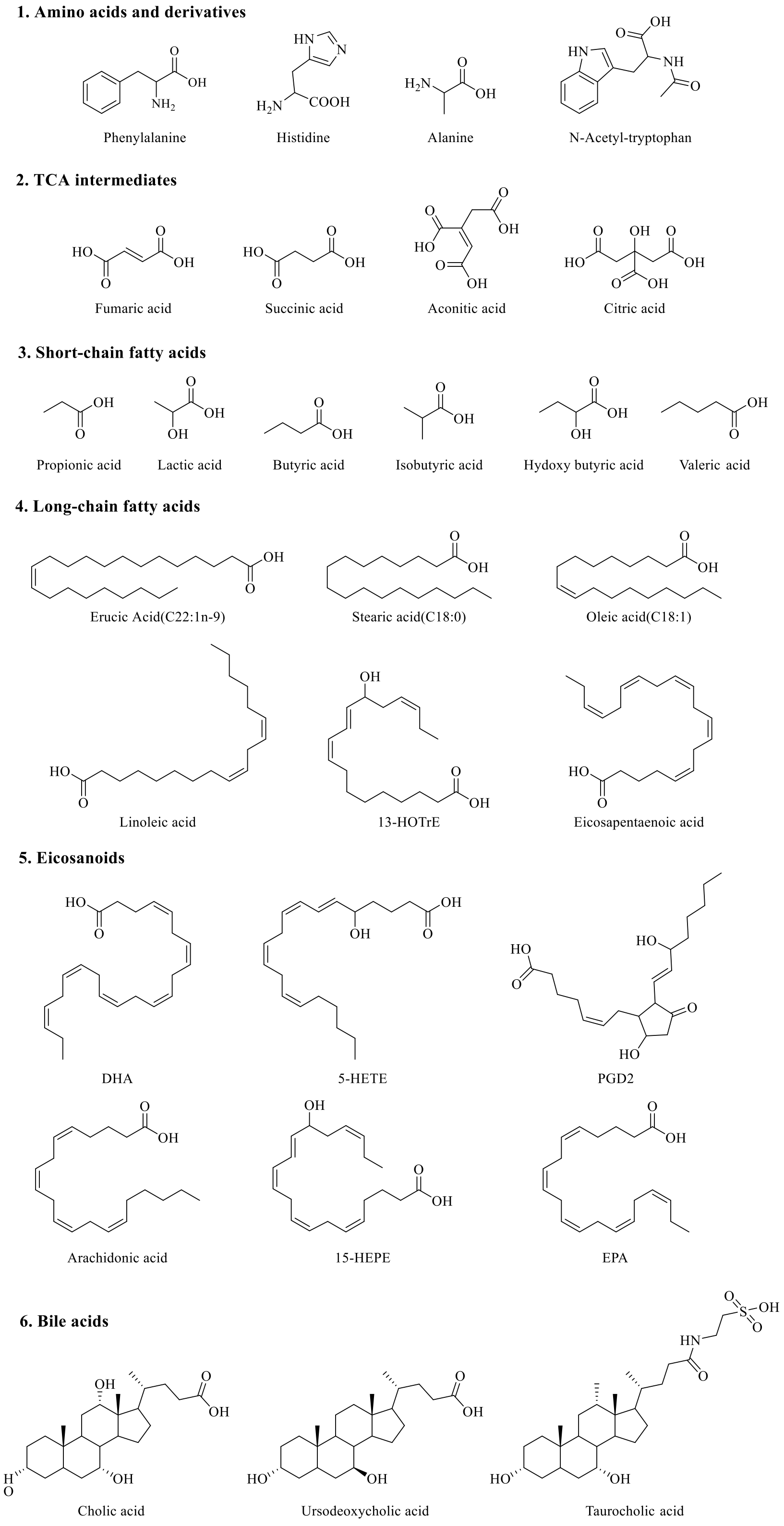

Figure S1 | Structures of representative carboxyl-containing metabolites in serum.

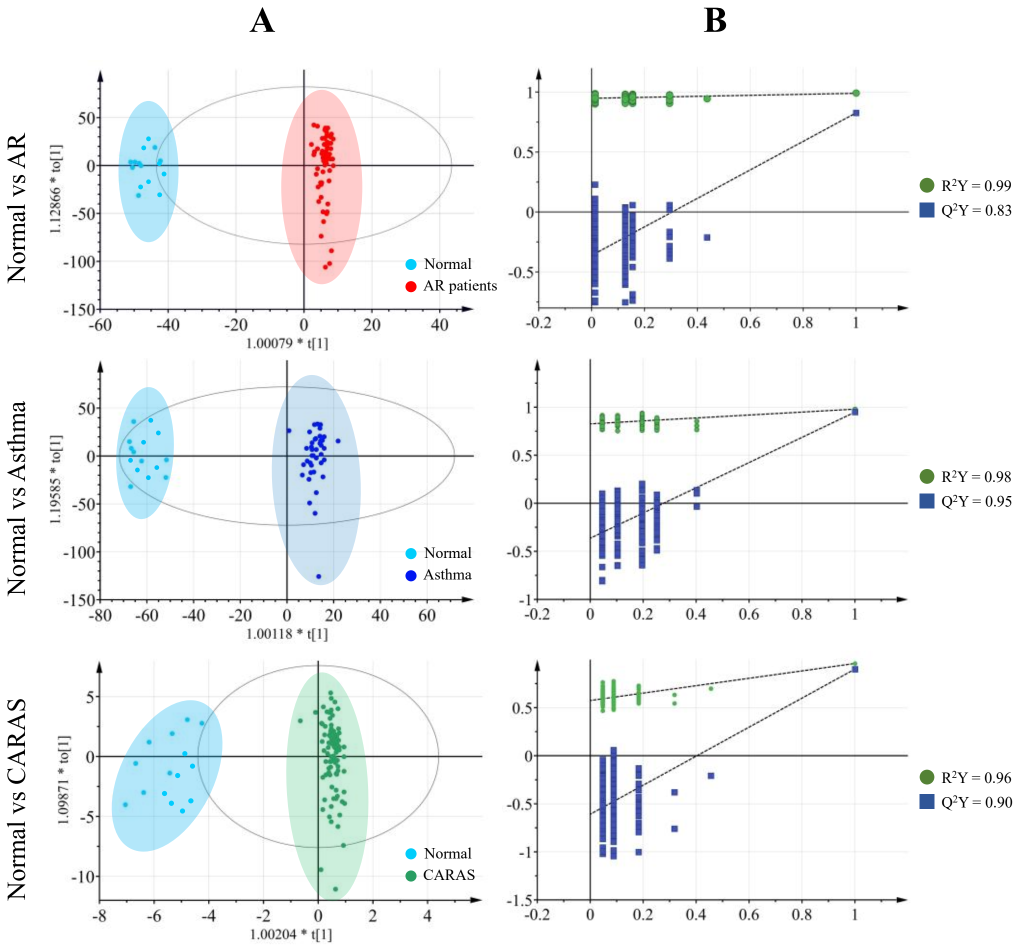

Figure S2 | Untargeted metabolomic analyses were performed using sera obtained from the discovery cohort. **(A)** OPLS-DA score plots for AR, CARAS and asthma group, compared to normal group. **(B)** 100 permutation tests to evaluate the quality of the OPLS-DA model.

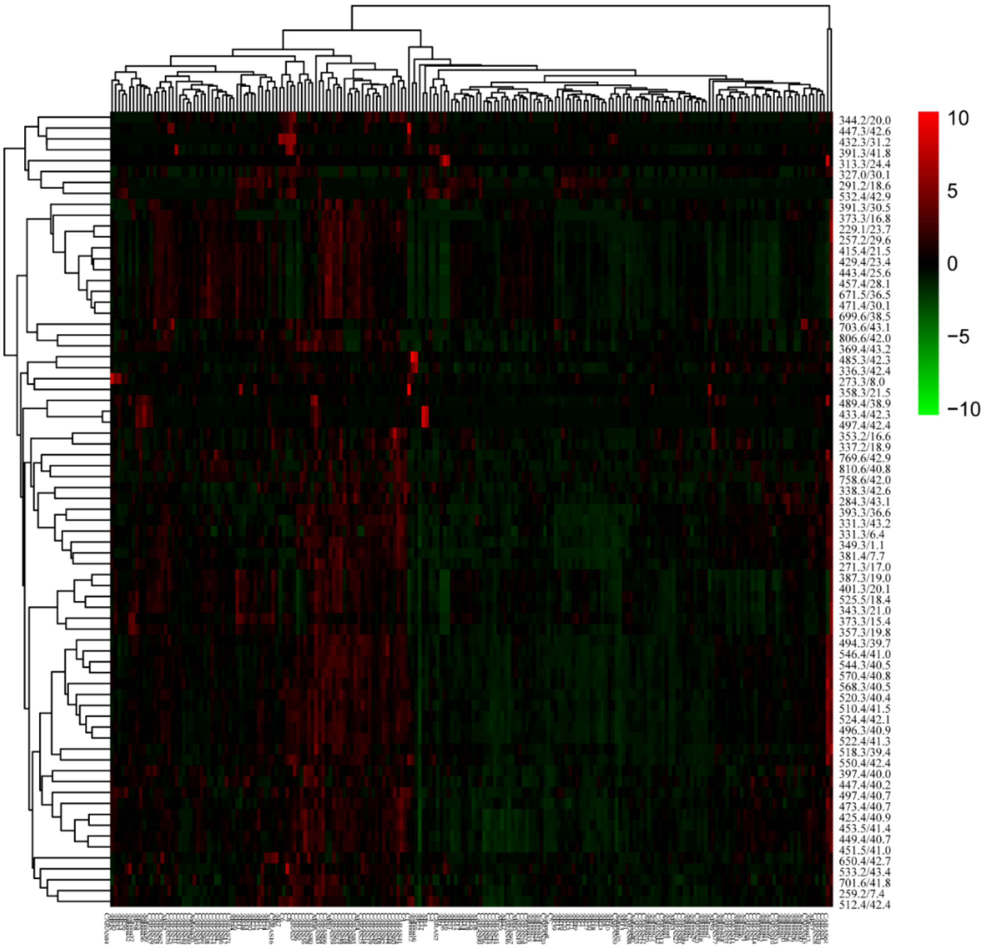

Figure S3 | Heatmap of 73 metabolites found in four different groups by untargeted metabolomics.

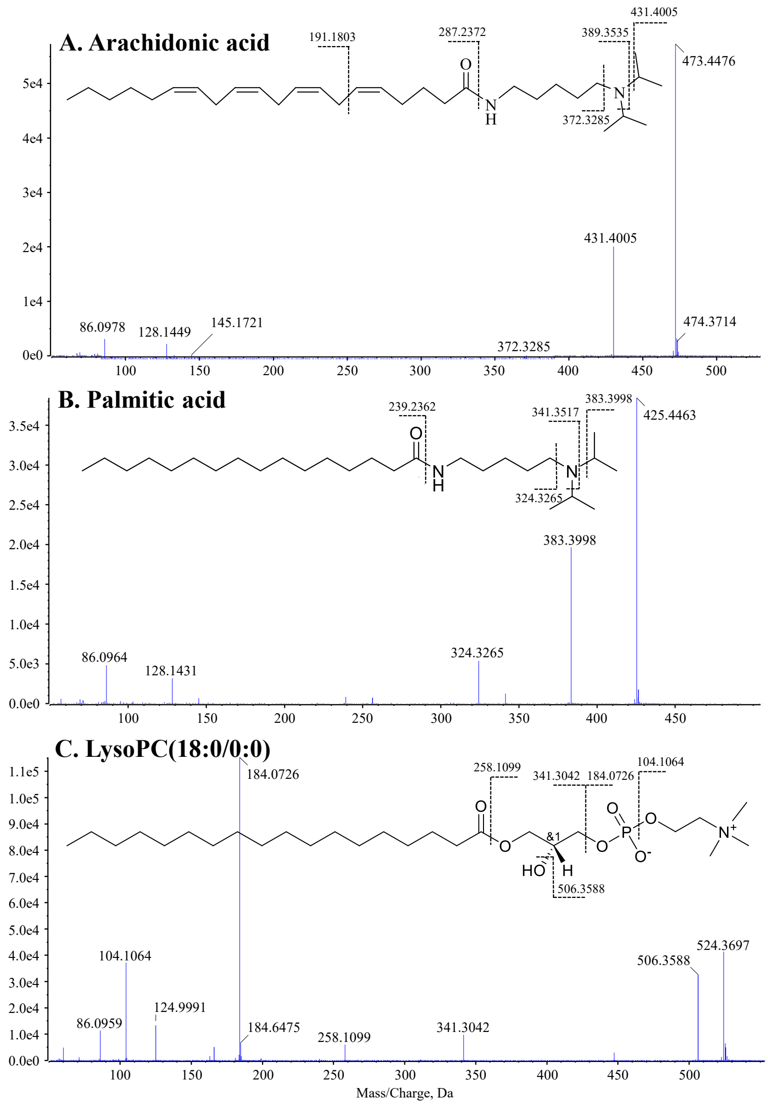

Figure S4 | MS/MS spectra of DIAAA-derivatized metabolites: arachidonic acid **(A)**, palmitic acid **(B)**, and non-derivatized metabolite: LysoPC(18:0/0:0) **(C)**.

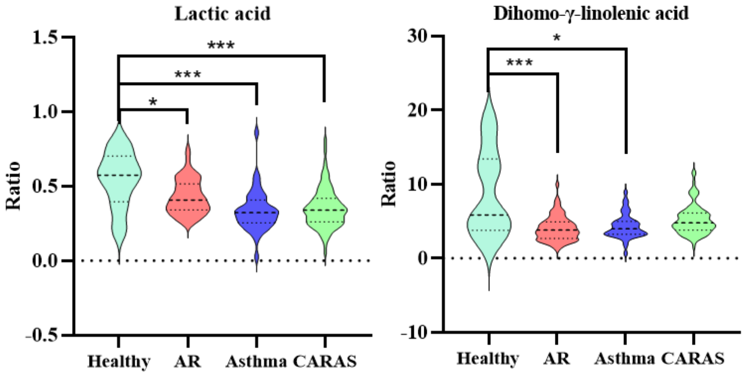

Figure S5 | Comparison of representative metabolites (lactic acid and dihomo-γ-linolenic acid) in children with AADs.

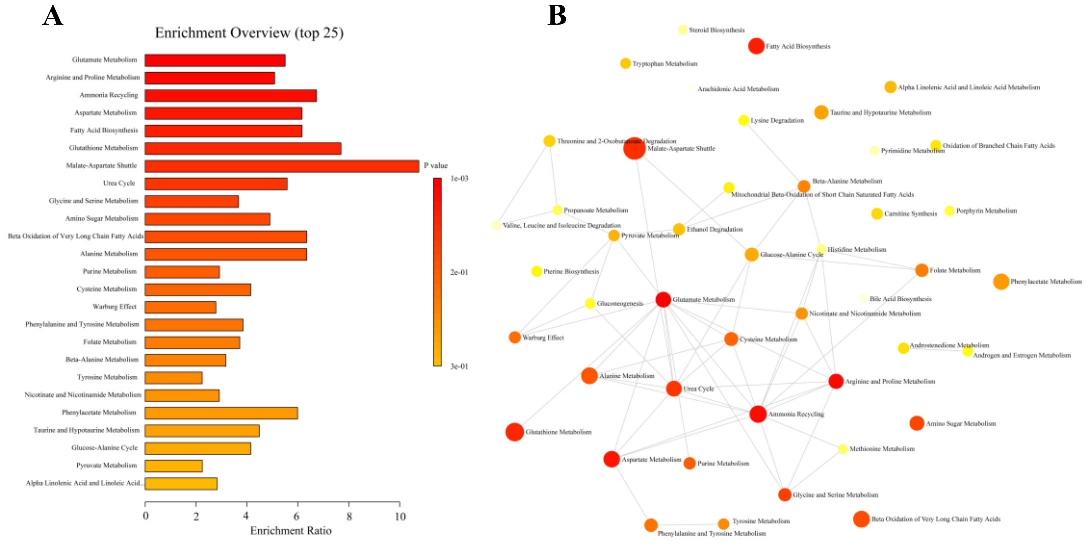

Figure S6 | **Pathway enrichment of the significant metabolites identified by untargeted and targeted approaches using the metabolic datasets. (A**) Barchart view, (**B**) network view.

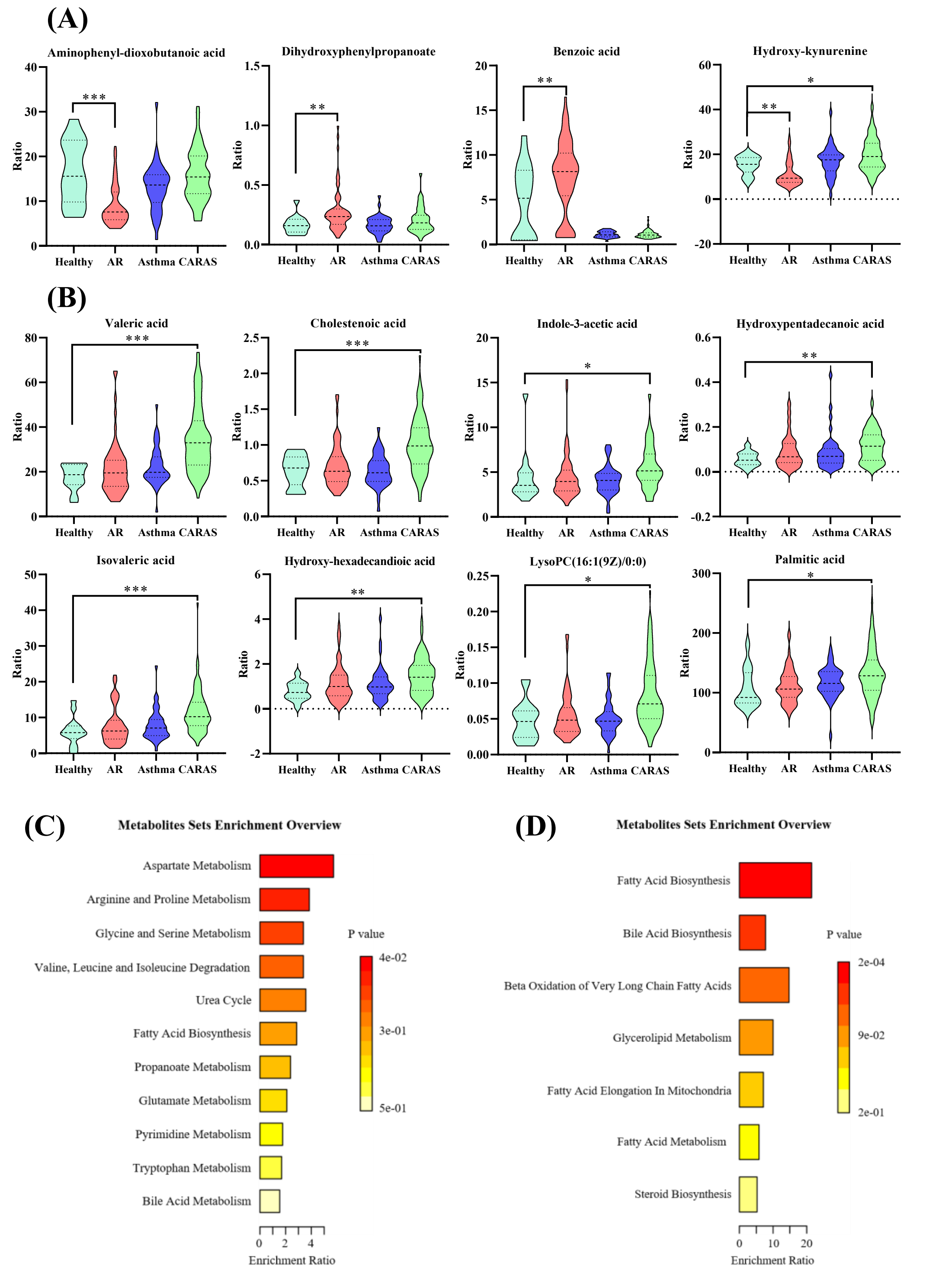

FIGURE S7 | Comparison of representative biomarker candidates specifically found in children with AR (**A**) and CARAS (**B**). *, **, *** denoted *P* <0.05, *P* <0.01, and *P* <0.001 in patients compared to healthy control. Metabolic pathway analysis based on differentially expressed metabolites in the AR (**C**) and CARAS (**D**).

Reference

[1] X. Bian, N. Li, B. Tan, B. Sun, M.Q. Guo, G. Huang, L. Fu, W.L.W. Hsiao, L. Liu, J.L. Wu, Polarity-Tuning Derivatization-LC-MS Approach for Probing Global Carboxyl-Containing Metabolites in Colorectal Cancer, Analytical chemistry, 90 (2018) 11210-11215.
